# Building shape-focused pharmacophore models for effective docking screening

**DOI:** 10.1186/s13321-024-00857-6

**Published:** 2024-08-09

**Authors:** Paola Moyano-Gómez, Jukka V. Lehtonen, Olli T. Pentikäinen, Pekka A. Postila

**Affiliations:** 1 MedChem.fi, Institute of Biomedicine, Integrative Physiology and Pharmacology, University of Turku, 20014 Turku, Finland; 2 InFLAMES Research Flagship, University of Turku, 20014 Turku, Finland; 3 Structural Bioinformatics Laboratory, Biochemistry, Faculty of Science and Engineering, Åbo Akademi University, 20500 Turku, Finland; 4 InFLAMES Research Flagship, Åbo Akademi University, 20500 Turku, Finland; 5 Aurlide Ltd, Lemminkäisenkatu 14A, 20520 Turku, Finland

**Keywords:** Distance-based graph clustering, Shape-focused pharmacophore modeling, Flexible-ligand molecular docking, Docking rescoring, Shape similarity, Virtual screening, Drug discovery, Benchmarking

## Abstract

**Abstract:**

The performance of molecular docking can be improved by comparing the shape similarity of the flexibly sampled poses against the target proteins’ inverted binding cavities. The effectiveness of these pseudo-ligands or negative image-based models in docking rescoring is boosted further by performing enrichment-driven optimization. Here, we introduce a novel shape-focused pharmacophore modeling algorithm O-LAP that generates a new class of cavity-filling models by clumping together overlapping atomic content via pairwise distance graph clustering. Top-ranked poses of flexibly docked active ligands were used as the modeling input and multiple alternative clustering settings were benchmark-tested thoroughly with five demanding drug targets using random training/test divisions. In docking rescoring, the O-LAP modeling typically improved massively on the default docking enrichment; furthermore, the results indicate that the clustered models work well in rigid docking. The C+ +/Qt5-based algorithm O-LAP is released under the GNU General Public License v3.0 via GitHub (https://github.com/jvlehtonen/overlap-toolkit).

**Scientific contribution:**

This study introduces O-LAP, a C++/Qt5-based graph clustering software for generating new type of shape-focused pharmacophore models. In the O-LAP modeling, the target protein cavity is filled with flexibly docked active ligands, the overlapping ligand atoms are clustered, and the shape/electrostatic potential of the resulting model is compared against the flexibly sampled molecular docking poses. The O-LAP modeling is shown to ensure high enrichment in both docking rescoring and rigid docking based on comprehensive benchmark-testing.

**Graphical Abstract:**

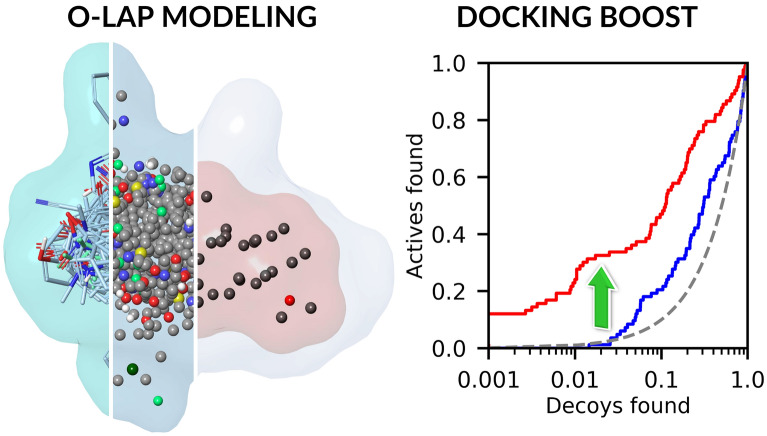

**Supplementary Information:**

The online version contains supplementary material available at 10.1186/s13321-024-00857-6.

## Introduction

Molecular docking is a structure-based drug discovery method applied routinely in massive virtual screening campaigns [1–3]. The main issue of docking is that while the flexible ligand sampling works acceptably [3–6], the docking scoring rarely works equally well or at all [5, 7–9]. This can render docking ineffective in practical drug discovery because active ligands are not enriched at the top of the ranking lists in large-scale virtual screening campaigns [10, 11]. Thus, costly physics-based post-processing [12–15], consensus docking [16–21], and alternative docking scoring or rescoring [22–24], such as machine learning-based scoring [25, 26], have been devised to improve the docking hit rates. The docking poses can also be filtered using ligand- and/or protein structure-based pharmacophore (PHA) models [27–29] or by applying specific protein-ligand interaction filters [30–33].

Ligand-based screening can rely on shape matching between the 3D template ligand and the screened compounds, and despite the simplicity of this approach, it often works better than docking in recognizing active ligands [34, 35]. For example, ROCS (Rapid Overlay of Chemical Structures; Open Eye Scientific Software) [36] or Shape-based Screening tool in Schrödinger’s MAESTRO [37] are widely used shape similarity comparison algorithms. ROCS-color estimates, in addition to the shape match, the chemical similarity of the superposed ligand groups using the Color Force Field [36]. ShaEP [38] is a non-commercial software option that can also be used to perform shape/electrostatic potential (ESP) similarity comparisons [38]. The only requirement for the shape-based screening is that an established active ligand and, preferably, its biologically relevant 3D conformer are known.

Although the shape match has traditionally been considered only between ligands, the ligand-protein cavity shape match is an inseparable and integral part of the molecular recognition process. Even in regular PHA modeling (e.g., LigandScout [39]), the shape matching between the screened ligand atoms and the PHA feature spheres can be applied together with indirect shape matching with the protein cavity via excluded protein volume. In the docking scoring, steric interactions with the protein are evaluated, but the overall shape match is not fully covered or emphasized by the point interaction-centered approach [36, 40]. While not directly applying the ligand-cavity shape match to virtual screening, several methods exist that evaluate the druggability of the protein cavities. This includes geometry-based (e.g., POVME [41–43], POCKET [44], PocketPicker [45], GHECOM [46]), energy-based (e.g., SiteMap [47], AutoLigand [48], Q-SiteFinder [49]), or data-driven (e.g., SCREEN [50], P2Rank [51], DeepPocket [52]) pocket detection methods.

A more direct drug discovery application of the ligand-cavity shape match is to use it in rigid docking, i.e., cavity-based negative images (e.g., SHAPE4 [53], SLIM [54], VOIDOO/FLOOD [55, 56], PANTHER [57]) are used as pseudo-ligand templates for shape similarity-based alignment and comparison [53, 54, 57–63]. In addition, it has been demonstrated that the shape/ESP features of PANTHER-generated NIB (negative image-based) models can be used effectively in docking rescoring [61, 64–68]. The NIB models are composed of neutral filler atoms and positively/negatively charged atoms that represent the protein cavity’s reciprocal H-bond donors and acceptors. The NIB models are directly compared against the flexibly sampled docking poses in a process known as negative image-based rescoring (R-NiB) [64, 67] using ShaEP [38]. The NIB model composition can be improved by incorporating atomic content from the protein structure-bound ligands [60] and, notably, by performing greedy search optimization known as brute force negative image-based optimization (BR-NiB) [65, 66].

**Fig. 1:**
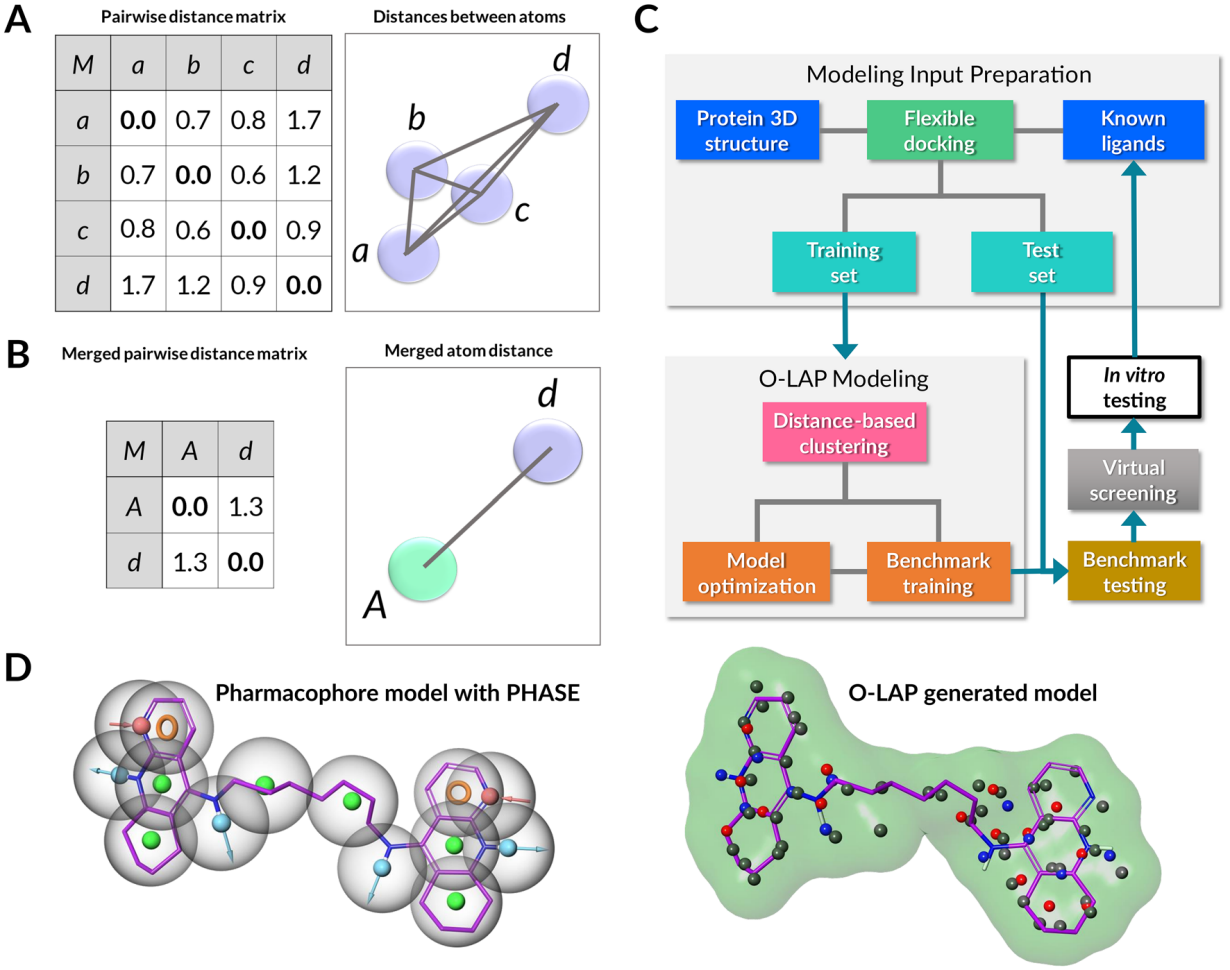
O-LAP graph clustering principle and workflow. **A** Before the graph clustering with O-LAP, all the pairwise distances (black lines) are measured between all atoms of the same atom type (N = 4; a-d; magenta discs). On the left, the pairwise distance matrix shows all the measured distances between the atoms. **B** After the graph clustering with O-LAP, three of the atoms within the search radii (a-c) are merged and, thus, given a new representative centroid atom (A; green disc). The resulting merged pairwise matrix is shrunk to contain only two atoms (A, d) and their distance. **C** In the workflow, the O-LAP modeling input originates from docking, and it is selected based on the original docking scoring. The O-LAP models are used to enrich docking-based virtual screening via shape similarity comparison before the in vitro testing, which in turn can result in the discovery of new hit compounds for generating more effective model versions. **D** An example of a typical PHA model, generated with PHASE [69] in MAESTRO2022-3, is shown for the acetylcholinesterase-bound inhibitor alkylene-linked tacrine dimer CHEMBL76173 (stick model with magenta backbone). The PHASE model contains PHA features such as H-bond donors/acceptors (blue outward arrow/red inward arrow), aromatic rings (orange ring), and hydrophobic groups (green spheres). The O-LAP model, which is composed of actual atoms filling the target’s cavity, focuses on shape matching instead of the specific PHA feature spheres (here all possible features shown) present in the equivalent PHASE model

In this study, it is shown that NIB-like cavity-filling or shape-focused PHA models (Fig. 1) can be generated relying solely on the protein-bound docked ligands. Firstly, the protein cavity is filled with flexibly docked active ligands. Secondly, the non-polar hydrogen atoms are trimmed, and covalent bonding information is deleted. Thirdly, the overlapping atoms with matching atom types are clumped together to form representative centroids by pairwise distance-based graph clustering (Fig. 1A, B). During clustering, atom-type-specific radii are applied in the distance measurements prior to the centroid generation. Fourthly, if a training set containing validated active ligands and inactive/decoy compounds is available, greedy search optimization can be performed to improve the model performance (Fig. 1C). In the end, the models can still contain a few overlapping atoms of different types, providing these clusters with a higher weight on the shape scoring than solitary atoms, but all in all, the process reduces the amount of redundant atomic input massively.

A new C++/Qt5-based algorithm, O-LAP, is presented for performing shape-focused PHA modeling. O-LAP is freely available for academic and commercial usage under GNU General Public License v3.0. Thorough testing was done with five benchmarking sets from the DUDE-Z database [70], which is an optimized version of DUD-E (A Database of Useful (Docking) Decoys–Enhanced) [71]. The results indicate that the O-LAP modeling (Fig. 1D) can improve the effectiveness of regular flexible molecular docking markedly, and it can even be used effectively in rigid docking. The shape-focused PHA models not only improve the performance of the docking algorithm PLANTS1.2 [72], but they often generate higher yields than the PANTHER-generated NIB models in rescoring usage. Several factors, such as the atomic input and clustering settings, affect the ultimate effectiveness of the method on a case-by-case basis.

In short, a new graph clustering software O-LAP is presented for generating shape-focused PHA models to facilitate effective docking-based virtual screening.

## Implementation

### Ligand and protein preparation

The modeling work was done using five DUDE-Z sets [70] (https://dudez.docking.org/; accessed in November 2021; Table S1), including neuraminidase (NEU) [73], A_2A_ adenosine receptor (AA2AR) [74], heat shock protein 90 (HSP90) [75], androgen receptor (AR) [76], and acetylcholinesterase (AChE) [77]. These sets with property-matched decoy compounds were selected because they have been found to be demanding not only for the docking scoring but also for the cavity shape-based rescoring [66] (Table S1). Although the ligand preparation was already done in a prior study [66], the general workflow is described below.

A pseudo-random number generator from the C++ standard library Mersenne Twister 19, 937 [72, 78] was used to generate the random 70/30 training/test set divisions (Table S1). LIGPREP in MAESTRO2017-1 (Schrödinger, LLC, New York, NY, USA, 2017) was used to generate 3D conformers from SMILES to MAE format and to add all tautomeric states and OPLS3 (Optimized Potentials for Liquid Simulations) partial charges. For the rigid docking, the alternative ligand 3D conformers were generated with CONFGENX in MAESTRO2022-3 (Schrödinger, LLC, New York, NY, USA, 2022). Before docking, the ligands were converted from MAE to MOL2 format using MOL2CONVERT in MAESTRO.

### Protein preparation and flexible molecular docking

The flexible-ligand docking was done in a prior study using PLANTS1.2 (http://www.tcd.uni-konstanz.de/plants_download/; Academic free license) [72] for all of the DUDE-Z sets except AChE [66]. The protein 3D structures, which were protonated using REDUCE3.24 (https://github.com/rlabduke/reduce/tree/master) [79], were acquired from the Protein Data Bank (PDB; https://www.rcsb.org/). The AChE docking was performed using a different PDB-entry (PDB: 2CKM) than in a previous study [77] to facilitate the binding of alkylene-linked tacrine dimers. The centroid of each co-crystallized ligand was used as a docking center with a box radius of 10 Å. Otherwise, the default settings of PLANTS, generating 10 binding predictions for each ligand, were applied.

### O-LAP model input preparation

50 top-ranked docked active ligands from the training set were extracted based on the ranking provided by the default PLANTS docking scoring function ChemPLP. The best-ranked pose (conf_01) for each ligand was selected into the input for the O-LAP modeling. Before the clustering, the non-polar hydrogen atoms of the docked ligands were removed, the separate MOL2 entries were merged and the covalent bonding data was removed. The O-LAP model dimensions could be limited by a 2.0 Å radius from the X-ray co-crystal ligand either before or after the O-LAP modeling in BODIL [80] (http://users.abo.fi/bodil/about.php).

### O-LAP: graph clustering principle

O-LAP, short for OVERLAP, is a C++/Qt5-based algorithm that is released under the GNU General Public License v3.0 via GitHub (https://github.com/jvlehtonen/overlap-toolkit). O-LAP builds the cavity-filling or shape-focused PHA models using any overlapping atomic content, such as protein-bound docked small-molecule ligands, for performing the shape/ESP-based docking rescoring or rigid docking using ShaEP (or similar methods). The input, given in the MOL2 format, contains the atom coordinates subjected to the clustering. O-LAP decreases the number of atoms by clustering the overlapping atoms of the same type and replacing them with representative centroid atoms. A cluster of overlapping atoms at the binding cavity gets replaced by a less cluttered cavity-filling model.

O-LAP performs distance-based graph clustering, in which atoms are seen as nodes that are subject to grouping based on relative pairwise distance measurements (Fig. 1A-B). O-LAP solves the nearest neighbor problem by systematically applying the atom type-specific search radii for each input atom. The radii are taken from the atom-specific bond lengths provided in the GAFF (General Amber Force Field) [81] and then reduced by 5%. However, the fixed value of 1.38 Å was applied to all aromatic atom types. During the clustering, pairwise distances are computed for all atoms belonging to the same type. If two or more identical atoms are within the same atom type-specific search radius, they are clumped together, and a new centroid atom is generated to represent them (Fig. 1A-B). The shortest distance pairs are considered before repeating the nearest neighbor distance check again for the other nearby atom pairs. The partial charge of the atom with the biggest charge difference against zero in a cluster is given for the new model atom.

In addition to the default clustering, MCL (Markov Clustering Algorithm; http://micans.org/mcl/; GNU General Public License v3.0) [78, 82–85] can also be used with the *--mcl* option provided that the external software is installed to the path and it is executable. MCL14.137 was used in the testing. The pairwise similarities of atoms of the same type are passed in the ABC format to MCL, which in turn performs the clustering unsupervised and automatically. The similarity for each atom pair is calculated with the Eq. 1

1 $$Similarity = MAX_{{{\text{distance}}}}^{2} - distance^{2}$$

where *MAX*_distance_ is the cutoff distance for the atom type.

### O-LAP: basic usage and the user-adjustable settings

The simplest usage case for O-LAP requires just typing in the executable and the input file containing the atomic input in the MOL2 format. For example, an O-LAP default model would be generated with the following command: “*o-lap input.mol2* > *output.mol2*”. It is, however, strongly recommended that the user experiments on at least a few alternative settings, to acquire more effective models.

The MCL clustering can be adjusted using the *--mclI* option (default 2), where the larger inflation values increase inequality by rescaling the distribution of transition probabilities in a way that preferred neighbors are further favored and less popular neighbors are demoted [86]. The MCL processing is fast, but the speed is reduced significantly if the inflation values below 2.0 are used (data not shown). The *--mclte* option can be used to perform the MCL clustering marginally faster with multiple threads. Regardless of the chosen clustering method, O-LAP operates very rapidly (Time = 0.03-30.5 ms; Table S2) with reasonably sized input (N = 916-1858; Table S2). However, the specific settings affect the time as well, for example, a higher* --clustermin* value can increase the processing time (Table S2).

The* --clustermin* option determines the minimum number of atoms for a cluster to be included in the output O-LAP model. For example, if a cluster contains two atoms of the same atom type and the minimum limit is set to three, the two-atom cluster would be discarded completely. By using this option, the model can be made to focus on those shape/ESP features that are shared between multiple closely aligned active ligands and, likewise, remove those outliers, where the docked ligand or some parts of it are outside the binding hotspot area. If the input contains atoms of a specific atom type that is deemed unnecessary (e.g., dummy atoms), they can be removed entirely using the *--deletetypes* < *str* > option, where they simply are given as a comma-separated list.

The* --nib* option can be used to make the O-LAP model more PANTHER/NIB model-like, i.e., all the clustered atoms are converted into positive N.3, negative O.3, or neutral C.3 or C.ar atom types based on their partial charges rather than the original atom type. The grouping into these four classes is done using the *--nibthreshold* < *num *> option, where the inputted value (default 0.2) can be set for making the partial charge-based classification. In this default scheme, the selected threshold values are given to the model atoms instead of the original partial charges (e.g., N.3 = 0.2, N.3 = -0.2, or C.3/C.ar = 0). The conversion of the atoms into the three NIB-like classes can also happen without changing the original partial charges of the input atoms using the *--nibcharged* option.

The *--clusterminchr* option sets the minimum size of the cluster for the charged atoms. Atom is regarded as charged if its partial charge exceeds the* --nibthreshold* value (default 0.2). This makes it possible to process charged atoms differently than the "neutral" atoms.

The *--cutoff* < *num* > option can be used to adjust the cutoff distance of all atoms not included in the default (or user-supplied) cutoff list. A user-specified set of cutoffs can be applied by inputting an alternative JSON file with the *--cutoffs* < *file/json* > option. The cutoff distances used in the clustering can be displayed in the terminal with the *--showcutoffs* option. The *--similar* option makes it possible to consider specific atom types as the same during the clustering, which can reduce the atom count of the output model. This approach can effectively remove overlapping atoms of different atom types provided that they are specifically listed as similar. An alternative list of similar atoms can be given in the JSON format using the *--similarjson* < *file* > option. The effective similar list can be displayed with *--showsimilar* option.

### Docking rescoring and rigid docking via shape similarity comparison

The shape/ESP-based similarity comparison was performed using a similarity comparison algorithm ShaEP1.3.1 (http://users.abo.fi/mivainio/shaep/) [38]. The docking rescoring was done using the *-noOpt* option, which prevents the algorithm from optimizing alignment or superposing the docked poses against the 3D template or O-LAP model. In contrast, during the rigid docking the *-noOpt* option was not applied and, thus, ShaEP was allowed to perform the coordinate transformations needed for acquiring the optimized alignment against the template. In the rigid docking, the ab initio-generated ligand 3D conformers were used in the similarity comparison instead of the flexibly docked poses. In the ShaEP scoring, the match between the template and the screened ligand ranges from 1 (perfect match) to 0 (no match at all) and, moreover, the default 50/50 shape/ESP weight ratio or shape alone (100/0) was used. Notably, ShaEP works with the Sybyl MOL2 atom typing which is also shared by PLANTS and O-LAP.

### O-LAP: clustering settings adjustment for assuring high enrichment

Several O-LAP settings were explored systematically for the five DUDE-Z targets using their respective training sets (Table S1) before the final testing (Table S3 vs. Table 1). Top-performing O-LAP settings for any target depend on multiple factors (Table S2) such as the underlying target protein 3D structure and the flexibly sampled docking poses or docking settings, the input atom composition, and the benchmark set composition. Thus, while the study does not provide default settings that would be guaranteed to work in every case, below are explained those O-LAP options or their combinations that one at least should consider.

**Table 1:** O-LAP modeling results for docking rescoring and rigid docking with the test sets.

Flexible-ligand molecular docking*					
Metrics	NEU	AA2AR	HSP90	AR	AChE
**AUC**	0.89 ± 0.04	0.72 ± 0.03	0.51 ± 0.06	0.60 ± 0.03	0.82 ± 0.02
**EFd 0.1%**	12	5.5	0	0	12
**EFd 0.5%**	24	14.1	0	0	31.6
**EFd 1.0%**	32	18.8	0	1.2	33.3
**EFd 5.0%**	56	35.2	4.8	12.5	47.9
**BR20**	0.54	0.34	0.06	0.12	0.54
**N atoms**	N/A	N/A	N/A	N/A	N/A

O-LAP models in docking rescoring					
**AUC**	***0.98*** ± ***0.02***	***0.78*** ± ***0.02***	***0.57*** ± ***0.07***	***0.80*** ± ***0.03***	***0.86*** ± ***0.02***
**EFd 0.1%**	***44***	***10.9***	***0***	***10***	***31.6***
**EFd 0.5%**	***64***	***17.2***	***0***	***15***	***37.6***
**EFd 1.0%**	***72***	***20.3***	***0***	***17.5***	***44.4***
**EFd 5.0%**	***88***	34.4	***33.3***	***32.5***	***59.8***
**BR20**	***0.83***	***0.35***	***0.27***	***0.36***	***0.64***
**N atoms**	54	54	67	57	72

O-LAP models optimized for docking rescoring					
**AUC**	***0.99*** ± ***0.01***	***0.79*** ± ***0.02***	***0.64*** ± ***0.07***	***0.83*** ± ***0.03***	***0.86*** ± ***0.02***
**EFd 0.1%**	***48***	***18.8***	***19***	***8.8***	***30.8***
**EFd 0.5%**	***64.0***	***32***	***23.8***	***20***	***40.2***
**EFd 1.0%**	***76***	***37.5***	***38.1***	***22.5***	***41***
**EFd 5.0%**	***100***	***48.4***	***42.9***	***40.0***	***60.7***
**BR20**	***0.91***	***0.49***	***0.45***	***0.40***	***0.66***
**N atoms**	47	28	34	49	51
**N generations**	8	26	33	8	21

Rigid docking for O-LAP models in docking rescoring					
**AUC**	***0.98*** ± ***0.02***	***0.74*** ± ***0.03***	0.48 ± 0.06	***0.81*** ± ***0.03***	***0.87*** ± ***0.02***
**EFd 0.1%**	***12***	***0.8***	***0***	***7.5***	***20.5***
**EFd 0.5%**	***52***	13.3	***0***	***12.5***	***36.8***
**EFd 1.0%**	***72***	17.2	***0***	***20***	***44.4***
**EFd 5.0%**	***92***	27.3	***14.3***	***42.5***	***65***
**BR20**	***0.84***	0.28	***0.11***	***0.39***	***0.67***
**N atoms**	54	60	61	57	63

The best results, improving or as good as the molecular docking, are shown in bold and italics. Here the EFd 0.1% and 0.5% were calculated for the first time for the original docking results (except for AChE) that were also published previously [66].

The *--clustermin* option (Fig. S1A) is worth exploring systematically alone or in combination with other options such as *--clusterminchr* (Fig. S1D) or *--mclI*. When used, it reduces the model’s atom count significantly as it removes non-common or outlier atom placements that are not shared by the other active ligands. In theory, it can exclude “bad”, rare, or inconsistent ligand poses or functional group placements from the cavity-filling input. For example, with NEU, the *--clustermin* values from 7 to 10 provided the highest enrichment factor improvement over docking (Fig. S1A). The usefulness of this option wanes when using too large values or when there are less ligand atoms to perform the clustering with.

The top-performing O-LAP models in this study were typically generated using MCL [86] instead of the default clustering method of O-LAP (Fig. 1A-B). However, the use of MCL alone was not enough to boost the enrichment to the highest levels, but the *--mclI* option had to be adjusted as well (Fig. S1D). The effective inflation values ranged from 5 to 20 for the five targets. The high* --mclI* values worked the best when combined with similarly high *--clustermin* values (Table S2). Moreover, the use of *--clusterminchr* (Fig. S1C) option resulted in satisfactory outcomes when it was used together with the *--clustermin* and the* --mclI* options. The operation of *--clusterminchr* option is shown at the atomic level for the Sybyl O.co2 atoms of NEU model in Fig. S2. Simply, by increasing the value from 2 to 8, reciprocally, the number of O.co2 atoms are gradually lowered from 13 to 2 (Fig. S2).

The *--similar* option, utilizing the default similar atom list, did not improve the model’s rescoring prowess, and its use is not recommended at least without careful adjustment. Likewise, the *--nib* option alone was not particularly useful, however when the option was paired with alternative values for *--nibthreshold*, *--clustermin,* or *--mclI*, it could sometimes excel. Due to the use of multiple altered settings at the same time, it is difficult to discern why the *--nib* option could sometimes improve the model composition, but it might be linked to altered vdW radii assisting in acquiring a more optimal shape match by chance. For getting the most NIB-like models for docking rescoring usage, one should revert to using PANTHER [57] instead of O-LAP.

Even the input models, containing just the merged ligands without the covalent bonds, can sometimes surpass the default docking enrichment in rescoring usage at least marginally (Table S4). However, because the resulting ShaEP scores are extremely low (Table S5), we do not recommend this elementary approach for any serious docking rescoring work. The sorting power of the unprocessed input is likely related to the cumulative weight of overlapping atoms at certain sections of the cavity rather than the shape scoring. Moreover, the O-LAP modeling was clearly needed for acquiring the highest enrichment values, especially for the very early enrichment. For example, the merged AChE model, containing ~2,000 atoms, beat docking with the training set in the rescoring with ShaEP (AUC: 0.81 ± 0.02 vs. 0.85 ± 0.01; Table S4), but the top-performing O-LAP model, containing only 72 atoms, did clearly better (AUC: 0.87 ± 0.01; Table S3). Lastly, after certain limit, the size of the model and/or ligand set starts to affect ShaEP computing efficiency negatively (data not shown).

### Optimization of O-LAP models via enrichment-driven greedy search

The atom compositions of O-LAP models were optimized using a greedy search method introduced in a prior study [66]. BR-NiB (brute force negative image-based optimization) was originally devised to improve the composition of PANTHER-generated NIB models; however, it applies to any kind of atomic data filling the target’s binding cavity. During the BR-NiB operation, the effect of each cavity atom on the model’s fitness or rescoring ability is tested systematically. Each atom is removed one by one from the model and the effects of these successive removals on the enrichment are evaluated by rescoring with the training set. The rescoring is done using ShaEP and the target enrichment metric or Boltzmann-enhanced discrimination of the receiver operating characteristic or BEDROC [87] with alpha value 20 (BR20) is calculated using ROCKER (https://www.medchem.fi/rocker/; MIT license) [88]. The new (-1 atom) model improving the rescoring performance the most is used as a template for next atom removals, rescoring, and enrichment evaluation until the yield improvement ends. The iterative sampling process, which is repetitive and not suitable for large models, does not represent an actual brute force approach as it only considers atom removals that improve the enrichment the most at each step. The Brutenib code is available online under the MIT license via GitHub (https://github.com/jvlehtonen/brutenib; MIT License).

### Figure preparation and analysis

The figures were generated using MAESTRO2022-3 and BODIL Modeling Environment [80]. The enrichment metrics and ROC (Receiver Operating Characteristics) curves were generated using ROCKER0.1.4 [88]. The overall enrichment was evaluated using the area under the curve (AUC) values and the Wilcoxon statistic [89, 90] was applied for the error estimation. The enrichment factors (or EFds) were calculated as a true positive rate in which 0.1%, 0.5%, 1.0%, and 5.0% of the decoy compounds were found. The BR20 values were also calculated to estimate early and overall enrichment. Tanimoto fingerprint similarity comparison was performed using CANVAS in MAESTRO2022-3 with the cutoff of 0.0 for compounds included at the top 1.0% of the rescored test set. This was done to determine if the O-LAP modeling was focusing the selection towards structurally similar ligands to the ones used as the clustering input.

## Results and discussion

### O-LAP: shape-focused pharmacophore modeling

In the PHA modeling (see, e.g., Fig. 1D), specific feature spheres are generated for matching functional groups that are found to overlap between multiple aligned or superposed compounds (e.g., docked ligands) [91–93]. In practice, a PHA model is a collection of these 3D features representing H-bond donors/acceptors, aromatic rings, hydrophobic or charged groups, etc. The validity of PHA models can improve when matches with active ligands are favored and, vice versa matches with inactive decoys are shunned. Although the O-LAP models are mainly used in docking rescoring in this study, the methodology can be perceived of as shape-focused PHA modeling, in which the flexible docking simply provides the ligand alignment.

In the O-LAP modeling, overlapping atoms filling the protein cavity are clumped together using atom type-specific and distance-based graph clustering. The attention stays firmly at the atomic level (Fig. 1A, B), where only the types, partial charges, and relative distances between atoms guide the automated graph clustering (Fig. 1D). This atomistic approach retains the all-encompassing shape component of the flexibly docked or otherwise protein-bound ligands [94] that is largely ignored in the traditional PHA modeling (Fig. 1D). Although unorthodox at first glance, the O-LAP modeling fits at least in part the PHA definition by the IUPAC (International Union of Pure and Applied Chemistry) [95] which defines it as "*the set of steric and electronic characteristics necessary to ensure optimal supramolecular interactions with a specific biological target structure and to trigger (or block) its biological response*".

Because the O-LAP modeling is based on molecular docking sampling, this assures direct involvement of the protein 3D structure in the ligand alignment. It also means that no geometry matches or alignment between the ligands were optimized for the input. As a result, the O-LAP modeling relies heavily on shape matching or steric interactions, although, in theory, ESP could play a bigger role depending on the specific ligand input. Regardless of the shape focus, even after clustering and optimization, the final O-LAP model can contain at least some overlapping atoms of different atom types, which, in turn, has the potential to enhance the weight of certain model sections in the ShaEP scoring.

In addition, the success of O-LAP modeling similarly relies on the quality of the input or training data as is the case with other PHA modeling methods. Experimentally established active ligands are needed as input for the model building (Fig. 1C; Table S1) and preferably inactive decoy compounds are also available for the enrichment-based settings adjustment or optimization. While not explored in this study, applying different docking algorithms (e.g., GLIDE, DOCK, AUTODOCK VINA, GOLD) [96–100] or alternative scoring functions (e.g., X-SCORE, ID-Score) [101, 102] as well as different benchmark-test sets (e.g., DUD, MUV, ULS/UDS) [71, 103, 104] or carefully selecting the protein 3D structure [33], both docking and O-LAP modeling could perform even better than reported here. In theory, even the initial ligand/pose alignment or selection does not have to rely on flexible-ligand docking as is the case with other PHA modeling methods.

### Docking scoring-based O-LAP models improve docking enrichment substantially

**Fig. 2:**
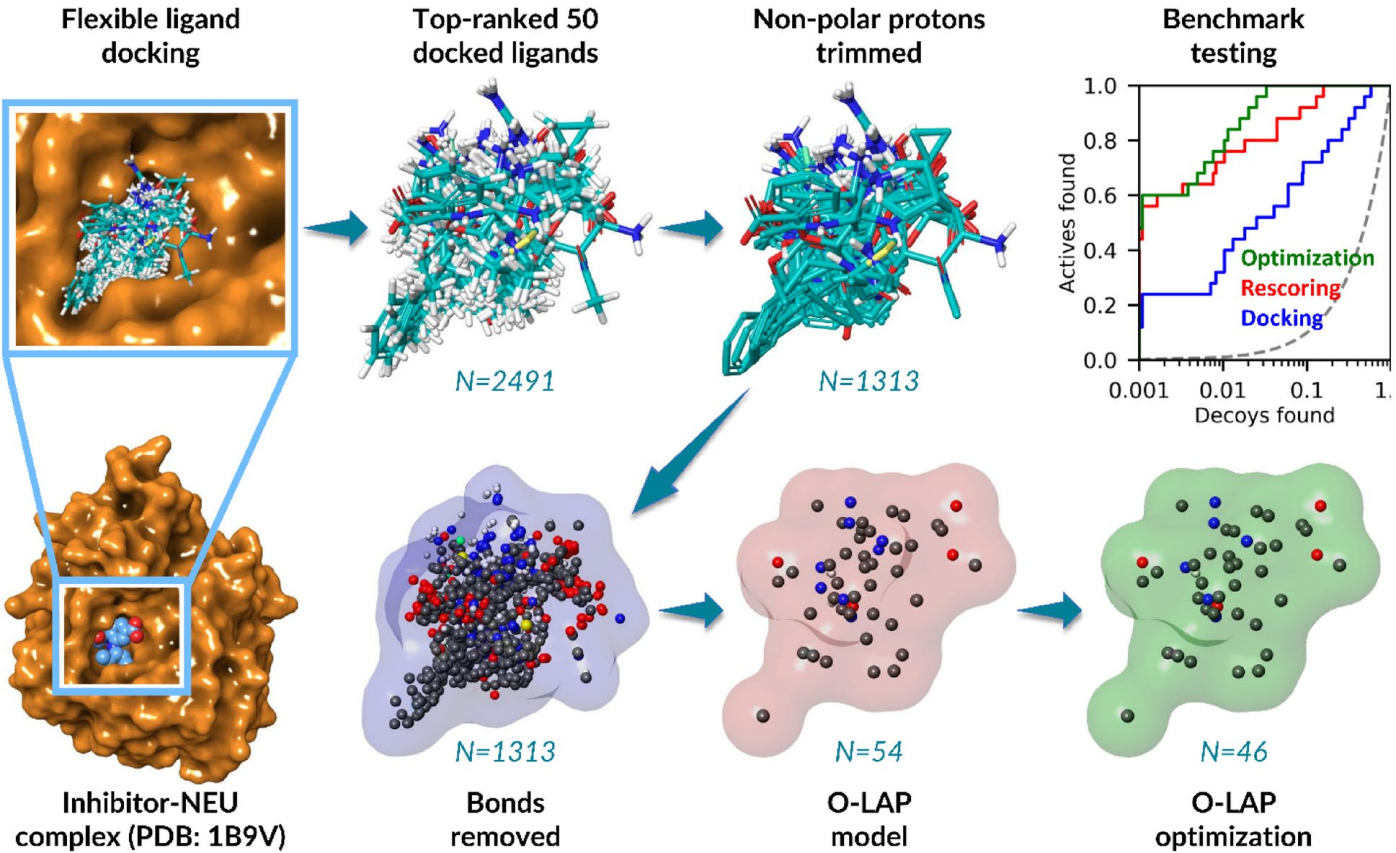
Docking scoring-based O-LAP modeling with neuraminidase. The ligands in the training set were flexibly docked into an X-ray crystal structure of NEU solved in complex with inhibitor BANA206 [73] (PDB: 1B9V; A chain; orange surface model). 50 active ligands ranked at the top by PLANTS (only best-ranked or conf_01 poses) were selected for the O-LAP modeling (stick models with blue carbons). Next, the non-polar hydrogen atoms (white stick models) were trimmed, covalent bonding information was removed, and the separate ligand entries were merged into a single MOL2 file (atoms shown as spheres; blue surface). The graph clustering with O-LAP generated a coherent model, where most of the overlapping and redundant atoms were clustered (red surface). The top-performing O-LAP model based on the training results worked similarly well in the benchmark-testing – the massive enrichment boost (Tables S3 and 1) in docking rescoring (red line) over the default docking (blue line) or random selection (dotted line) is visible in the semilogarithmic ROC curves. The greedy search optimization of the model (green surface) improved the rescoring enrichment marginally (green line). Multiple O-LAP options were explored but only the top-performing model settings are shown in Table S2

In the training, the top-performing O-LAP model could always improve on the default docking scoring function of PLANTS [72], when it was generated using the docking scoring-based input (Table S3). This indicates that PLANTS could find reasonable or at least consistently similar poses of the active ligands for the O-LAP modeling and shape/ESP-based rescoring (Fig. 2). Importantly, this enrichment improvement was also seen with the randomly selected test sets for all targets (Table 1).

The default docking did well with the NEU if considering either the AUC, BR20, or early enrichment values, such as the EFd 1.0% (Fig. 2; Table 1). Regardless, the O-LAP modeling improved the docking enrichment on all these metrics – the boost was even statistically significant for the AUC value that jumped from 0.89 ± 0.04 to 0.98 ± 0.02 (Table 1). Impressively, the EFd 1.0% of docking was improved from 32 to 72 –the massive boost is visible in the semilogarithmic ROC curves as well (Fig. 2). Although the enrichment boost for the AA2AR was modest, it could be seen with most of the calculated metrics, moreover, it was again statistically significant for the AUC value. With the HSP90 and AChE, the O-LAP modeling made notable or at least modest improvements compared to the default docking. For example, the AUC value of HSP90 increased from 0.51 ± 0.06 to 0.57 ± 0.07 and, likewise, with the AChE the same metric jumped from 0.82 ± 0.02 to 0.86 ± 0.02. Finally, with the AR, the O-LAP modeling was especially effective on the early enrichment as indicated by the EFd 1.0% value which improved from 1.2 to 17.5 (Table 1).

These positive results (Fig. 2; Table 1) indicate that the docking scoring-based input works well in the O-LAP modeling. The approach is dependent on using active ligands as input, as the clustering of docked inactive ligands did not generate effective models (data not shown) and, furthermore, it is crucial that the correctness of the input poses is carefully estimated before performing the clustering.

### Greedy search optimization boosts O-LAP modeling enrichment massively

The greedy optimization with the BR-NiB approach has been shown to work excellently with the PANTHER-generated NIB models and the co-crystal-NIB hybrid models in the past [65, 66, 68]. The BR-NiB method has already been used successfully in the docking-based virtual screening for retinoic acid-related orphan receptor γt modulators [68]. However, applying the optimization directly to massive input or models containing hundreds or even thousands of overlapping atoms is too time-consuming or computationally costly. By performing the O-LAP modeling before running the parallelized processing, the optimization of enormous input becomes suddenly feasible (Fig. 2); for example, an optimization of an HSP90 input of 665 atoms (Table S4), that would take approximately one month to process using 18 CPUs, is performed in ~30 min when paired up with the O-LAP modeling (Tables 1 and S3; from 54 to 33 atoms).

When optimized, the O-LAP models improve on the enrichment of flexible-ligand docking massively. Notably, with the docking scoring-based O-LAP models, the optimization of the NEU model acquired an impressive AUC value of 0.99 ± 0.01 and almost as impressive BR20 value of 0.91 (Table 1; Fig. 2). For the HSP90, the optimized O-LAP model improved the enrichment on every metric compared to the non-optimized model; for example, EFd 1.0% value jumped from 0 to 38.1 (Table 1). If compared to the cavity-based BR-NiB results from our prior study [66], the optimized O-LAP models typically did better in the docking rescoring than the optimized NIB models.

### Rigid docking with the O-LAP models outperforms the default flexible docking

**Fig. 3:**
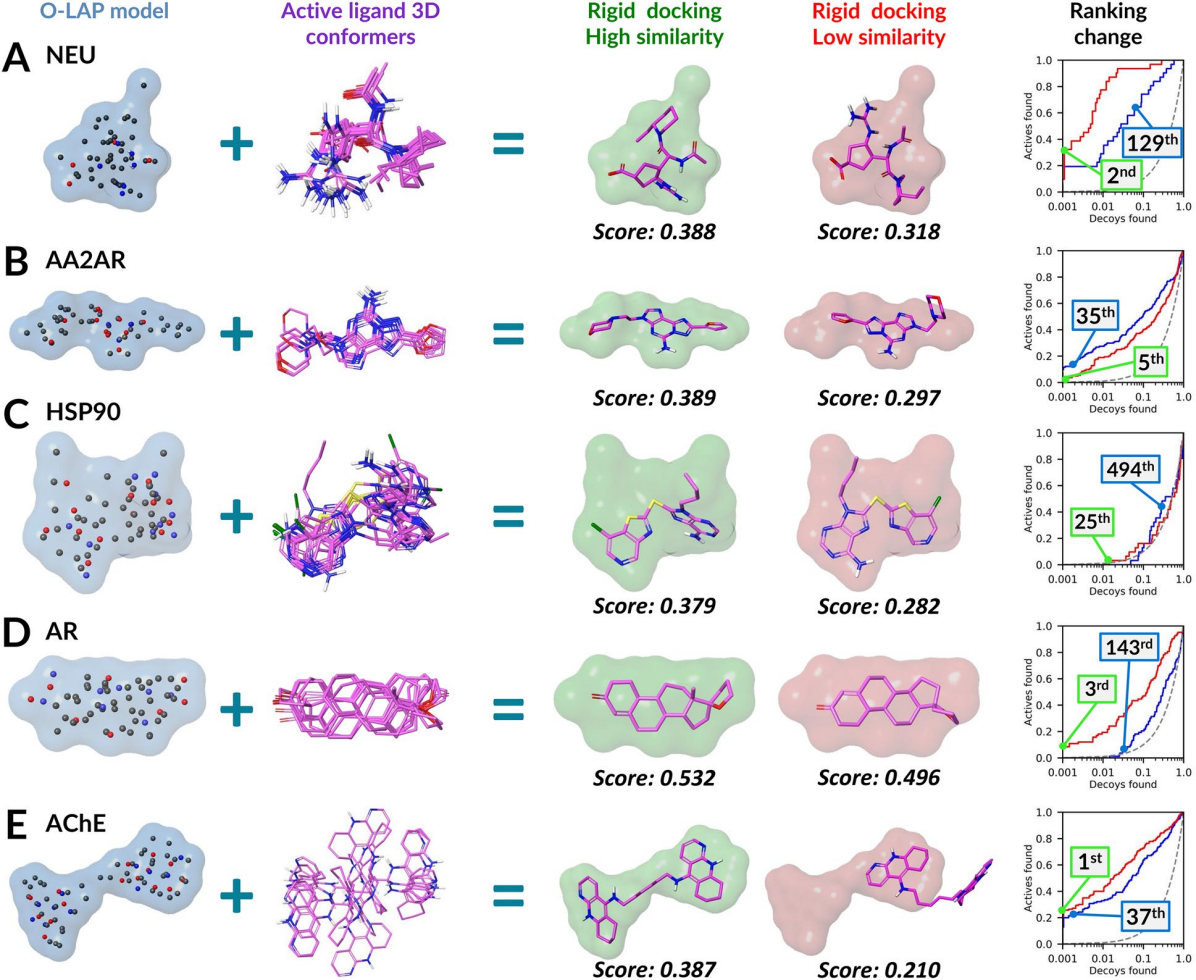
Examples of O-LAP models promoting the discovery of active ligands in rigid docking. The active CHEMBL ligand conformation with the best rigid docking result (green surface) and the worst conformation (red surface) are shown with the actual ShaEP scores. **A** NEU with CHEMBL294169 (129^th^ → 2^nd^); **B** AA2AR with CHEMBL1088236 (35^th^ → 5^th^); **C** HSP90 with CHEMBL377958 (494^th^ → 25^th^); **D** AR with CHEMBL75050 (143^th^ → 3^rd^); and **E** AChE with CHEMBL75305 (37^th^ → 1^st^). In the rigid docking, the O-LAP model (red line) boosts the default docking enrichment (blue line) for all targets (apart from AA2AR and HSP90) based on the semi-logarithmic ROC curves. For further information see Fig. 2

The cavity-based NIB models were initially intended for rigid docking known as NIB screening [57, 58, 62]. Likewise, the O-LAP models can also be used in rigid docking (Fig. 3). In fact, the O-LAP-based rigid docking generated higher enrichment than the default docking scoring of PLANTS with all targets in the testing, apart from the AA2AR and HSP90 (Table 1; Fig. 3). If comparing the ranking positions of active ligands from the O-LAP-based rigid docking against the PLANTS flexible docking (Table S6), the best ranking improvements in favor of rigid docking are highlighted in the ROC curves (right panel in Fig. 3A-E). Notably, with the AChE, HSP90, and NEU, even the ligand-based screening done with the co-crystal ligands as templates (Table S1) did better than the flexible docking alone (Table 1 vs. Table S7), indicating the challenging nature of the DUDE-Z sets for the standard docking method [70].

Regardless, the O-LAP models did far worse in the rigid docking than when they were applied to rescoring flexibly sampled docking poses (Table 1). The lower rigid docking performance was expected as the methodology is coarser regarding the sampling than the flexible-ligand docking. Moreover, only a decent shape match is needed for effective rescoring (Table S8), but, vice versa, the ESP matching plays a bigger role in the rigid docking as it affects the H-bonding and, ultimately, the ligand placement directly. Moreover, the O-LAP settings adjustment using training sets for rigid docking takes far more time than what is the case for rescoring. If this computing cannot be done, the O-LAP models that performed well in docking rescoring did also reasonably well in the rigid docking (data not shown).

### O-LAP focuses on high-quality binding predictions of docking

**Fig. 4:**
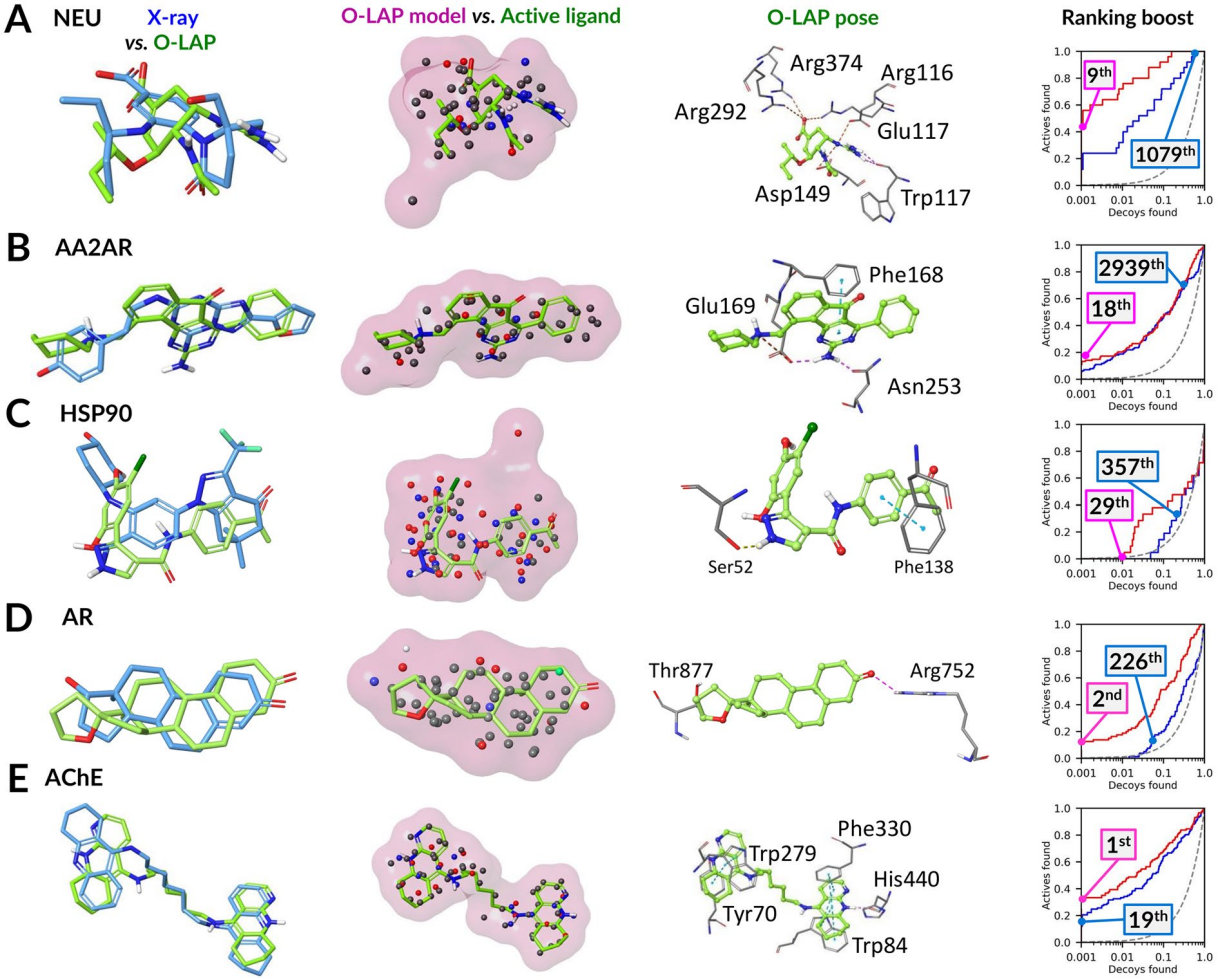
Examples of O-LAP models promoting the discovery of active ligands in docking rescoring. The O-LAP rescoring (red line) boosts the default docking (blue line) enrichment for all targets (marginal improvement for AA2AR) based on the semi-logarithmic ROC curves. The boost was most notable for the following active CHEMBL ligands (green stick models): **A** CHEMBL311059 with NEU (1079^th^ → 9^th^); **B** CHEMBL1087820 with AA2AR (2939^th^ → 18^th^); **C** CHEMBL386399 with HSP90 (357^th^ → 29^th^); **D** CHEMBL312500 with AR (226^th^ → 2^nd^); and **E** CHEMBL76173 with AChE (19^th^ → 1^st^). The shape match between the top poses and the O-LAP models (pink transparent surface) is evident when inspecting the ligand-model overlays. The docking poses for the active ligands are also comparable to the co-crystallized ligands (blue stick models; Table S1) included in the protein structures applied in the flexible docking. Although these active ligands differ in their chemical composition, there exist clear similarities in the key functional group placements. For further information see Fig. 2

The ranking boost of the O-LAP modeling compared to docking was excellent for the individual active ligands ranked at the top (Table S9). The active ligands with the largest ranking boosts were examined in detail for the NEU (1079^th^ → 9^th^; Fig. 4A), AA2AR (2939^th^ → 18^th^; Fig. 4B), HSP90 (357^th^ → 29^th^; Fig. 4C), AR (226^th^ → 2^nd^; Fig. 4D), and AChE (19^th^ → 1^st^; Fig. 4E). The poses that O-LAP modeling promoted for these particular ligands had clearly matching functional group placements inside the cavity with the co-crystallized ligands. Note, that despite the similarity, the compared compounds were chemically different. The ligand-model shape match is excellent, and, moreover, there exist several well-coordinated ligand-protein interactions, such as π-π stacking or H-bonding, justifying their high-ranking positions. If simply ordering the top 20 compounds based on the original docking positioning, one would have missed these particular compounds and their likely correct or biologically relevant poses altogether. This is a well-documented shortcoming of the default docking scoring [72, 105, 106], although, in this regard, PLANTS is a top-notch option among its peers [26, 107, 108]. In addition, based on Tanimoto fingerprint similarity comparison, the O-LAP models did not overly focus the compound selection towards chemically similar ligands to the input (Table S10).

### Shape matching provides the ranking boost

The partial charges of the input ligand atoms are varied and, importantly, this charge component is also retained in the generated O-LAP models. The ESP can be used along the shape similarity when screening is performed with ShaEP. However, the results indicate that the ESP scoring is low compared to the shape scoring, and the combined 50/50 shape/ESP scoring is about half of the shape score (Table S8). This indicates that the O-LAP models, prepared using the docking scoring-based input, do not possess optimal charge distribution either for docking rescoring or rigid docking.

Given these results for the ESP similarity, the O-LAP modeling results were also reweighted using only the shape score of ShaEP. In the rescoring usage, the shape-only approach for the O-LAP modeling always provided better results than the default docking scoring both in training (Table S3 vs. Table S11) and testing (Table 1 vs. Table 2). With the HSP90 and AA2AR, the shape only approach did marginally better than the default 50/50 approach (Tables 2 and S11), but, generally, the removal of ESP did not really affect the results significantly. In the rigid docking, the shape only approach generated better or as good results as the flexible docking; the only exception being the AA2AR (Table 2 and S11). Due of this almost singular focus on shape, the O-LAP modeling method is referred to as shape-focused PHA modeling, but this focus could change with different input.

**Table 2:** O-LAP modeling results in docking rescoring and rigid docking with the test sets based on shape-only matching.

Shape only O-LAP models in docking rescoring					
Metrics	NEU	AA2AR	HSP90	AR	AChE
**AUC**	***0.95* ± *0.03***	***0.78* ± *0.02***	***0.60* ± *0.07***	***0.81* ± *0.03***	***0.86* ± *0.02***
**EFd 0.1%**	***44***	***14.1***	***0***	***8.8***	***30.8***
**EFd 0.5%**	***56***	***18***	***9.5***	***13.8***	***37.6***
**EFd 1.0%**	***68***	***21.1***	***9.5***	***21.2***	***45.3***
**EFd 5.0%**	***80***	***35.2***	***28.6***	***28.8***	***60.7***
**BR20**	***0.80***	***0.36***	***0.28***	***0.33***	***0.64***
**N atoms**	58	54	45	48	72

Rigid docking for Shape only O-LAP models in docking rescoring					
**AUC**	***0.96* ± *0.03***	***0.68* ±*0.03***	***0.46* ± *0.06***	***0.80* ± *0.03***	***0.87* ± *0.02***
**EFd 0.1%**	***16***	***7.8***	***0***	***2.5***	***20.5***
**EFd 0.5%**	***32***	13.3	***0***	***12.5***	***35.9***
**EFd 1.0%**	***60***	17.2	***4.8***	***16.2***	***42.7***
**EFd 5.0%**	***88***	28.1	***19***	***35***	***64.1***
**BR20**	***0.80***	0.28	***0.13***	***0.35***	***0.65***
**N atoms**	70	46	61	88	61

The best results, improving or as good as the molecular docking, are shown in bold and italics. Here the EFd 0.1% and 0.5% were calculated for the first time for the original docking results (except for AChE) that were also published previously [66].

## Conclusions

This study presents a new shape-focused pharmacophore (PHA) modeling method and algorithm O-LAP (short for OVERLAP). The software can be used to generate cavity-filling or shape-focused PHA models using flexibly docked ligands. Massive amounts of atomic data with repetitive and overlapping content are untangled and clumped together using ultra-fast pairwise distance-based graph clustering. A seemingly “messy” cluster of atoms at the binding site is streamlined into a coherent cavity-filling or shape-focused PHA model matching roughly the shape or steric contours of the protein’s binding cavity. The shape/ESP comparison of the O-LAP models against the flexibly docked ligands in the docking rescoring or even in rigid docking can be performed using existing similarity comparison algorithms such as ShaEP. Thorough benchmark-testing indicates that O-LAP models are highly suitable for rescoring flexibly sampled docking poses – the default docking enrichment is massively improved with five targets using random training/test set divisions. O-LAP is available free for both academic and commercial usage under the GNU General Public License v3.0 via GitHub (https://github.com/jvlehtonen/overlap-toolkit).

## Availability and requirements

Project name: OVERLAP (O-LAP). Project home page: https://github.com/jvlehtonen/overlap-toolkit. Operating system: Platform independent (tested on Linux). Programming language(s): C++/Qt5. Other requirements: None. License: GNU GLPv3. Any restrictions to use by non-academic: None.

### Supplementary Information

Additional file1

Additional file2

## Data Availability

The datasets supporting the conclusions of this article are included in the supplementary_files.zip.
