# Supplementary material for: Building shape-focused pharmacophore models for effective docking screening
Source: J Cheminform. 2024 Aug 9;16:97. doi: 10.1186/s13321-024-00857-6 (PMC11312248; doi:10.1186/s13321-024-00857-6)
Supplement: Supplementary file 1 — Additional file1 [file 13321_2024_857_MOESM1_ESM.docx]

**Supporting Information**

**for**

**Building Shape-Focused Pharmacophore Models for Effective Docking Screening**

Paola Moyano-Gómez ^1,2^, Jukka V. Lehtonen ^3,4^, Olli T. Pentikäinen ^1,2,5^, Pekka A. Postila ^1,2,5,*^

^1^ MedChem.fi, Institute of Biomedicine, Integrative Physiology and Pharmacology, University of Turku, FI-20014 Turku, Finland

^2^ InFLAMES Research Flagship, University of Turku, 20014 Turku, Finland

^3^ Structural Bioinformatics Laboratory, Biochemistry, Faculty of Science and Engineering, Åbo Akademi University, FI-20500 Turku, Finland

^4^ InFLAMES Research Flagship, Åbo Akademi University, FI-20500 Turku, Finland

^5^ Aurlide Ltd., FI-20500 Turku, Finland

^*^ Correspondence: [pekka.postila@utu.fi](mailto:pekka.postila@utu.fi)


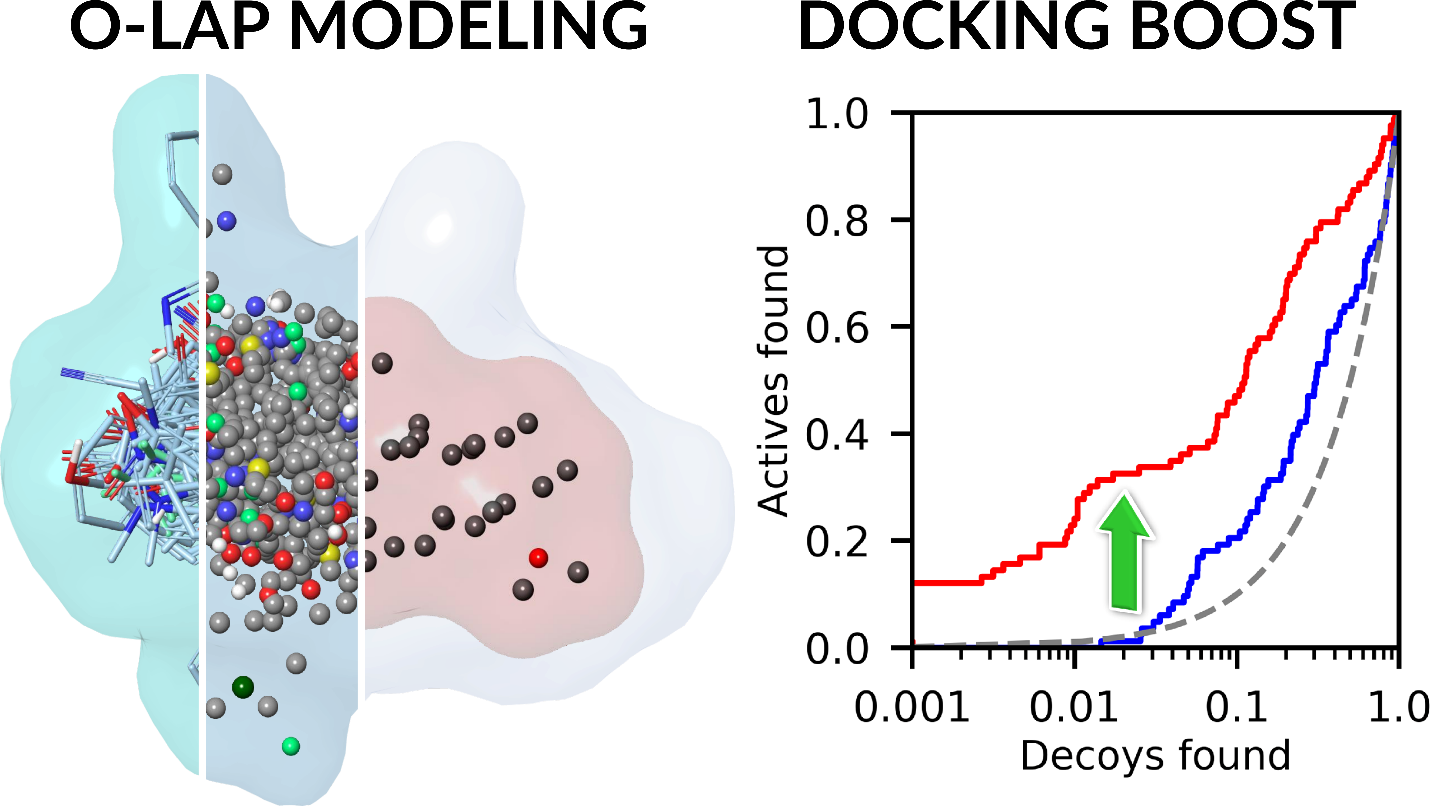


**Table S1**. DUDE-Z benchmarking sets and target protein 3D structures

| **Target Protein ^(1)^** | **PDB code** | **Resolution (Å)** | **Mechanism of action** | **Chain** | **Co-crystal** | **No. of active ligands ^(2)^** | **No. of inactive decoys ^(2)^** |
| --- | --- | --- | --- | --- | --- | --- | --- |
| NEU | 1B9V | 2.35 | inhibitor | A | RA2 | 66/25 | 4270/1830 |
| AA2AR | 3EML | 2.60 | antagonist | A | ZMA | 352/128 | 20402/2868 |
| HSP90 | 6LTK | 2.14 | inhibitor | A | E0G | 67/21 | 3990/1710 |
| AR | 2AM9 | 1.64 | agonist | A | TES | 151/80 | 6904/4123 |
| AChE | 2CKM | 2.15 | inhibitor | A | A77 | 300/117 | 6690/2868 |

^(1)^ Target proteins: neuraminidase (NEU) [1], A2A adenosine receptor (AA2AR) [2], heat shock protein 90 (HSP90) [3], androgen receptor (AR) [4], and acetylcholinesterase (AChE) [5].

^(2)^ The number (No.) of DUDE-Z [6] active ligands and inactive decoy molecules after the implementation of the ligand preparation with LIGPREP in MAESTRO 2017-1 (Schrödinger, LLC, New York, NY, USA, 2017).

**Table S2**. The applied O-LAP settings for the best models in the docking rescoring and rigid docking usage with the training sets.

| ***Docking Rescoring*** | **Docking scoring-based models** | | | | | **Shape only docking scoring-based models** | | | | |
| --- | --- | --- | --- | --- | --- | --- | --- | --- | --- | --- |
| **Settings** | **NEU** | **AA2AR** | **HSP90** | **AR** | **AChE** | **NEU** | **AA2AR** | **HSP90** | **AR** | **AChE** |
| ***ldlim*** | *no* | *after* | *no* | *no* | *before* | *after* | *after* | *no* | *no* | *before* |
| ***ldlim (Å)*** | - | 2 | - | - | 2 | 2 | 2 | - | - | 2 |
| ***--clustermin*** | 5 | 1 | 8 | 6 | 7 | 4 | 1 | 8 | 6 | 7 |
| ***--clusterminchr*** | - | - | - | 5 | - | 5 | - | - | - | - |
| ***--nib*** | - | yes | yes | - | yes | - | yes | - | - | yes |
| ***--nibthreshold*** | - | 0.55 | 0.1 | - | 0.25 | - | 0.55 | - | - | 0.25 |
| ***--mcl*** | yes | yes | - | yes | - | yes | yes | yes | yes | - |
| ***--mclI*** | 20 | 2 | - | 3.5 | - | 20 | 2 | 2.5 | 3.5 | - |
| **N atoms** | 54 | 54 | 67 | 57 | 72 | 58 | 54 | 45 | 48 | 72 |
| **Time (s)** | 0.265 | 0.434 | 0.069 | 0.136 | 11.910 | 0.160 | 0.477 | 0.209 | 0.155 | 10.325 |
| **Input** | 1313 | 1278 | 1508 | 916 | 1858 | 1082 | 1278 | 1508 | 916 | 1858 |
| ***Rigid Docking*** | **Docking scoring-based models** | | | | | **Shape only docking scoring-based models** | | | | |
| ***ldlim*** | *no* | *after* | *no* | *no* | *before* | *after* | *before* | *no* | *no* | *before* |
| ***ldlim (Å)*** | *-* | *2* | *-* | - | *2* | *2* | *2* | *-* | - | 2 |
| ***--clustermin*** | *5* | *1* | *8* | *5* | *7* | *3* | 3 | *8* | *4* | *8* |
| ***--clusterminchr*** | *5* | *-* | *-* | - | - | *5* | *-* | - | *-* | - |
| ***--nib*** | *-* | *yes* | *yes* | *yes* | *yes* | - | *yes* | *yes* | - | *yes* |
| ***--nibthreshold*** | *-* | *0.45* | *0.30* | *0.25* | *0.30* | - | *0.6* | *0.3* | - | *0.3* |
| ***--mcl*** | *yes* | *yes* | *-* | - | - | *yes* | *-* | *-* | *yes* | *-* |
| ***--mclI*** | *20* | *-* | *-* | - | - | *20* | *-* | *-* | 5 | *-* |
| **N atoms** | *54* | *60* | *61* | *57* | *63* | *70* | *46* | *61* | *88* | *61* |
| **Time (s)** | 0.256 | 0.540 | 5.744 | 2.487 | 23.308 | 0.181 | 12.609 | 5.744 | 0.182 | 24.757 |
| **Input** | 1313 | 1278 | 1508 | 916 | 1858 | 1082 | 1278 | 1508 | 916 | 1858 |

The settings that are underlined and shown in italics correspond to those models that differ from the top-performing model for the training set in the docking rescoring with ShaEP. The ligand distance limit (--*ldlim*) was used to limit the O-LAP input (*before*) or output model (*after*) to a radius of 2 Å from the co-crystallized ligand.

**Table S3**. O-LAP modeling results for docking rescoring and rigid docking with the training set.

| **Flexible-ligand molecular docking*** | | | | | |
| --- | --- | --- | --- | --- | --- |
| **Metrics** | **NEU** | **AA2AR** | **HSP90** | **AR** | **AChE** |
| **AUC** | 0.92±0.02 | 0.74±0.02 | 0.63±0.04 | 0.57±0.02 | 0.81±0.02 |
| **EFd 0.1%** | 12.1 | 11.7 | 0 | 0 | 9.7 |
| **EFd 0.5%** | 24.2 | 18.9 | 1.5 | 2.3 | 20.7 |
| **EFd 1.0%** | 30.3 | 22 | 1.5 | 2.8 | 27 |
| **EFd 5.0%** | 56.1 | 34.9 | 6 | 11.9 | 48 |
| **BR20** | 0.53 | 0.36 | 0.07 | 0.12 | 0.51 |
| **O-LAP models in docking rescoring** | | | | | |
| **AUC** | ***0.98±0.01*** | ***0.79±0.01*** | ***0.77±0.03*** | ***0.82±0.02*** | ***0.87±0.01*** |
| **EFd 0.1%** | ***54.5*** | *11.1* | ***3*** | ***11.9*** | ***28.3*** |
| **EFd 0.5%** | ***77.3*** | ***19*** | ***17.9*** | ***17.9*** | ***31.7*** |
| **EFd 1.0%** | ***81.8*** | ***24.7*** | ***25.4*** | ***23.2*** | ***39.3*** |
| **EFd 5.0%** | ***90.9*** | ***36.9*** | ***41.8*** | ***44.4*** | ***64.7*** |
| **BR20** | ***0.88*** | ***0.38*** | ***0.41*** | ***0.43*** | ***0.66*** |
| **N atoms** | 54 | 54 | 67 | 57 | 72 |
| **O-LAP models optimized for docking rescoring** | | | | | |
| **AUC** | ***0.99±0.01*** | ***0.81±0.01*** | ***0.82±0.03*** | ***0.84±0.02*** | ***0.87±0.01*** |
| **EFd 0.1%** | ***59.1*** | ***18.5*** | ***23.9*** | ***9.3*** | ***25.3*** |
| **EFd 0.5%** | ***80.3*** | ***33*** | ***44.8*** | ***19.9*** | ***37.7*** |
| **EFd 1.0%** | ***87.9*** | ***38.4*** | ***49.3*** | ***26.5*** | ***44.7*** |
| **EFd 5.0%** | ***93.9*** | ***52.3*** | ***68.7*** | ***50.3*** | ***67.3*** |
| **BR20** | ***0.92*** | ***0.51*** | ***0.65*** | ***0.48*** | ***0.69*** |
| **N atoms** | 47 | 28 | 34 | 49 | 51 |
| **N generation** | 8 | 26 | 33 | 8 | 21 |
| **Rigid docking for O-LAP models in docking rescoring** | | | | | |
| **AUC** | ***0.99±0.01*** | ***0.77±0.01*** | ***0.66±0.04*** | ***0.84±0.02*** | ***0.87±0.01*** |
| **EFd 0.1%** | ***45.5*** | *4.3* | ***0*** | ***5.9*** | ***22.3*** |
| **EFd 0.5%** | ***68.2*** | 12.5 | ***1.5*** | ***13.3*** | ***27.7*** |
| **EFd 1.0%** | ***89.4*** | 16.2 | ***6*** | ***21.3*** | ***32.7*** |
| **EFd 5.0%** | ***95.5*** | 28.1 | ***35.8*** | ***46.8*** | ***56.7*** |
| **BR20** | ***0.91*** | 0.30 | ***0.28*** | ***0.44*** | ***0.60*** |
| **N atoms** | 54 | 60 | 61 | 57 | 77 |

The best results, improving or as good as the molecular docking, are shown in bold and italics. Here the EFd 0.1% and 0.5% were calculated for the first time for the original docking results (except for AChE) that were also published previously [7].

**Table S4**. Direct rescoring with the input before O-LAP modeling with the training sets.

| **No ligand distance limit applied ^(1)^** | | | | | |
| --- | --- | --- | --- | --- | --- |
| **Metrics** | **NEU** | **AA2AR** | **HSP90** | **AR** | **AChE** |
| **AUC** | ***0.96±0.02*** | ***0.79±0.01*** | ***0.70±0.04*** | ***0.80±0.02*** | ***0.85±0.01*** |
| **EFd 0.1%** | 4.5 | *5.4* | ***0*** | ***3.3*** | ***22.3*** |
| **EFd 0.5%** | ***39.4*** | *18.8* | ***1.5*** | ***9.9*** | ***30*** |
| **EFd 1.0%** | ***56.1*** | ***23.3*** | ***1.5*** | ***11.9*** | ***35.3*** |
| **EFd 5.0%** | ***83.3*** | 34.1 | 4.5 | ***29.1*** | ***57.3*** |
| **BR20** | ***0.74*** | 0.35 | ***0.08*** | ***0.30*** | ***0.60*** |
| **N atoms** | 1313 | 1581 | 1508 | 916 | 2049 |
| **Ligand distance limit applied ^(2)^** | | | | | |
| **AUC** | ***0.96±0.02*** | ***0.75±0.02*** | ***0.70±0.04*** | ***0.80±0.02*** | ***0.85±0.01*** |
| **EFd 0.1%** | ***10.6*** | 1.7 | 0 | ***4.6*** | ***23.7*** |
| **EFd 0.5%** | ***40.9*** | 8.5 | 0 | ***9.9*** | ***30.7*** |
| **EFd 1.0%** | ***50*** | 13.6 | 0 | ***13.2*** | ***35.7*** |
| **EFd 5.0%** | ***83.3*** | 29.3 | ***7.5*** | ***29.8*** | ***59.3*** |
| **BR20** | ***0.72*** | 0.28 | ***0.08*** | ***0.31*** | ***0.62*** |
| **N atoms** | 1082 | 1278 | 1275 | 867 | 1858 |

^(1)^ No ligand distance limit from the co-crystal ligand was applied from the O-LAP input.

^(2)^ Ligand distance limit radius of 2.0 Å from the co-crystal ligand was applied from the O-LAP input.

The best results, improving or as good as the molecular docking, are shown in bold and italics. Here the EFd 0.1% and 0.5% were calculated for the first time for the original docking results (except for AChE) that were also published previously [7].

**Table S5**. The ShaEP scoring of the input prior to O-LAP modeling with the training set.

| **Ligand type** | **Shape/ESP weight** | **Metric** | **NEU** | **AA2AR** | **HSP90** | **AR** | **AChE** |
| --- | --- | --- | --- | --- | --- | --- | --- |
| Decoys | 50/50 | **AVG** | 0.020 | 0.018 | 0.022 | 0.028 | 0.014 |
|  |  | **MIN** | 0.012 | 0.005 | 0.009 | 0.010 | 0.002 |
|  |  | **MAX** | 0.028 | 0.026 | 0.029 | 0.039 | 0.027 |
|  |  | **MED** | 0.022 | 0.019 | 0.020 | 0.026 | 0.012 |
|  | 100/0 | **AVG** | 0.041 | 0.035 | 0.043 | 0.055 | 0.027 |
|  |  | **MIN** | 0.025 | 0.010 | 0.017 | 0.021 | 0.004 |
|  |  | **MAX** | 0.056 | 0.052 | 0.058 | 0.079 | 0.053 |
|  |  | **MED** | 0.043 | 0.038 | 0.041 | 0.052 | 0.023 |
|  | 0/100 | **AVG** | 2.28E-68 | 2.25E-41 | 1.28E-39 | 1.41E-33 | 3.94E-51 |
|  |  | **MIN** | 2.17E-82 | 3.84E-57 | 7.03E-46 | 2.61E-58 | 2.18E-95 |
|  |  | **MAX** | 3.22E-65 | 4.67E-37 | 2.29E-36 | 2.43E-30 | 2.28E-47 |
|  |  | **MED** | 5.68E-73 | 5.38E-55 | 2.05E-41 | 3.80E-54 | 4.77E-71 |
| Actives | 50/50 | **AVG** | 0.025 | 0.020 | 0.023 | 0.031 | 0.019 |
|  |  | **MIN** | 0.020 | 0.013 | 0.019 | 0.022 | 0.007 |
|  |  | **MAX** | 0.028 | 0.025 | 0.027 | 0.039 | 0.027 |
|  |  | **MED** | 0.026 | 0.021 | 0.022 | 0.035 | 0.020 |
|  | 100/0 | **AVG** | 0.050 | 0.040 | 0.046 | 0.061 | 0.039 |
|  |  | **MIN** | 0.041 | 0.025 | 0.038 | 0.043 | 0.014 |
|  |  | **MAX** | 0.056 | 0.049 | 0.054 | 0.077 | 0.053 |
|  |  | **MED** | 0.052 | 0.042 | 0.044 | 0.070 | 0.040 |
|  | 0/100 | **AVG** | 2.32E-68 | 1.69E-47 | 2.93E-40 | 3.20E-36 | 6.80E-73 |
|  |  | **MIN** | 2.17E-82 | 6.13E-56 | 1.16E-44 | 1.19E-56 | 3.62E-94 |
|  |  | **MAX** | 3.22E-65 | 2.82E-45 | 8.54E-39 | 2.05E-34 | 8.31E-71 |
|  |  | **MED** | 9.06E-79 | 4.38E-53 | 3.91E-44 | 1.18E-50 | 2.45E-75 |

The ShaEP [8] scores of the top-performing O-LAP models with the training set are shown as average (AVG), minimum (MIN), maximum (MAX), and median (MED) values. The scoring is provided for both inactive decoys and active ligands using the shape only (100/0), ESP only (0/100), and equal weight shape/ESP (50/50) values.

**Table S6**. Active ligand ranking changes due to rigid docking using the top docking scoring-based O-LAP models with the test sets.

| **NEU** | | **AA2AR** | | **HSP90** | | **AR** | | **AChE** | |
| --- | --- | --- | --- | --- | --- | --- | --- | --- | --- |
| **CHEMBL** | **Ranking** | **CHEMBL** | **Ranking** | **CHEMBL** | **Ranking** | **CHEMBL** | **Ranking** | **CHEMBL** | **Ranking** |
| 136111 | 4 → 1 | 248299 | 35 → 5 | 377958 | 494→25 | 377958 | 61→2 | 75305 | 37 → 1 |
| 294169 | 129 → 2 | 469876 | 62 → 6 | 448474 | 250→65 | 448474 | 143→3 | 522286 | 11 → 2 |
| 57800 | 91 → 4 | 1088236 | 74 → 10 | 386399 | 361→79 | 386399 | 227→4 | 1084775 | 26 → 3 |
| 195871 | 3 ← 6 | 360166 | 10 ← 14 | 377631 | 135→112 | 377631 | 1813→5 | 484705 | 72 → 4 |
| 436250 | 353 → 7 | 85864 | 41 → 15 | 178130 | 485→132 | 178130 | 3216→7 | 76658 | 19 → 5 |
| 400552 | 1 ← 8 | 275636 | 28 → 18 | 208239 | 259←280 | 208239 | 825→9 | 1086007 | 8 → 6 |
| 426810 | 8 ← 9 | 1088247 | 152 → 22 | 207307 | 254←292 | 207307 | 4165→11 | 257126 | 30 → 7 |
| 195288 | 503 → 10 | 274274 | 91 → 23 | 371915 | 165←376 | 371915 | 110→14 | 1172618 | 1 ← 8 |
| 292393 | 128 → 11 | 179644 | 5 ← 30 | 215657 | 277←395 | 215657 | 3839→15 | 50031 | 6 ← 9 |
| 350298 | 55 → 12 | 1093480 | 30 ← 34 | 200469 | 322←427 | 200469 | 3959→20 | 384886 | 176 → 10 |
| 164976 | 604 → 14 | 246262 | 764 → 35 | 426446 | 617→552 | 426446 | 1732→34 | 175555 | 3 ← 11 |
| 301435 | 182 → 15 | 471974 | 16 ← 36 | 212850 | 84←691 | 212850 | 3242→35 | 478667 | 13 → 12 |
| 25549 | 25 → 18 | 1087462 | 2281 → 39 | 377371 | 129←714 | 377371 | 1013→36 | 367067 | 2 ← 13 |
| 81286 | 61 → 20 | 373560 | 46 → 40 | 200628 | 459←813 | 200628 | 483→42 | 1173761 | 4 ← 14 |
| 442774 | 23 ↔ 23 | 426952 | 1899 → 45 | 399530 | 181←835 | 399530 | 926→46 | 225077 | 27 → 15 |
| 278852 | 92 → 25 | 472460 | 7347 → 49 | 467399 | 1391→873 | 467399 | 3566→55 | 370807 | 15 ← 16 |
| 423292 | 67 → 27 | 184309 | 143 → 57 | 514748 | 915←1039 | 514748 | 4158→70 | 266045 | 360 → 17 |
| 1161284 | 271 → 28 | 469893 | 17 ← 62 | 209356 | 1700→1168 | 209356 | 1158→71 | 15056 | 41 → 18 |
| 318452 | 46 → 29 | 601628 | 1771 → 63 | 517583 | 1132←1196 | 517583 | 2546→77 | 490866 | 7 ← 19 |
| 228008 | 188 → 32 | 1087819 | 2482 → 65 | 366215 | 514←1245 | 366215 | 3894→78 | 76173 | 23 → 20 |

The O-LAP rigid docking could generate higher (→), lower (←) or the same (**↔;** e.g., 442774 for NEU**)** ranking for the individual active ligands (CHEMBL codes) than the flexible docking performed with PLANTS. For brevity, only the first 20 top-ranked active ligands are shown.

**Table S7**. X-ray co-crystals in rigid docking with test sets.

| **Metrics** | **NEU** | **AA2AR** | **HSP90** | **AR** | **AChE** |
| --- | --- | --- | --- | --- | --- |
| **AUC** | ***0.92±0.04*** | 0.63±0.03 | ***0.54±0.06*** | 0.48±0.03 | ***0.85±0.02*** |
| **EFd 0.1%** | ***64*** | 0 | ***9.5*** | ***0*** | ***23.1*** |
| **EFd 0.5%** | ***76*** | 1.6 | ***9.5*** | ***1.2*** | 28.2 |
| **EFd 1.0%** | ***76*** | 4.7 | ***9.5*** | ***1.2*** | ***40.2*** |
| **EFd 5.0%** | ***84*** | 18.8 | ***9.5*** | 6.2 | ***58.1*** |
| **BR20** | ***0.83*** | 0.16 | ***0.13*** | 0.07 | ***0.61*** |
| **N atoms** | 77 | 27 | 36 | 22 | 77 |
| **Ligand** | *RA2* | *ZMA* | *E0G* | *TES* | *AA7* |

The best results, improving or as good as the molecular docking, are shown in bold and italics. Here the EFd 0.1% and 0.5% were calculated for the first time for the original docking results (except for AChE) that were also published previously [7].

**Table S8**. The ShaEP scoring of the best O-LAP rescoring models with the test sets.

| **Ligand type** | **Shape/ESP weight** | **Metric** | **NEU** | **AA2AR** | **HSP90** | **AR** | **AChE** |
| --- | --- | --- | --- | --- | --- | --- | --- |
| Decoys | 50/50 | **AVG** | 0.268 | 0.301 | 0.295 | 0.297 | 0.237 |
|  |  | **MIN** | 0.178 | 0.056 | 0.142 | 0.148 | 0.070 |
|  |  | **MAX** | 0.336 | 0.386 | 0.379 | 0.343 | 0.372 |
|  |  | **MED** | 0.285 | 0.308 | 0.307 | 0.303 | 0.279 |
|  | 100/0 | **AVG** | 0.533 | 0.601 | 0.590 | 0.594 | 0.471 |
|  |  | **MIN** | 0.348 | 0.111 | 0.284 | 0.296 | 0.140 |
|  |  | **MAX** | 0.671 | 0.772 | 0.758 | 0.686 | 0.743 |
|  |  | **MED** | 0.569 | 0.615 | 0.613 | 0.605 | 0.559 |
|  | 0/100 | **AVG** | 6.65E-03 | 8.93E-04 | 5.18E-04 | 1.99E-05 | 3.61E-03 |
|  |  | **MIN** | 5.82E-07 | 3.24E-07 | 1.19E-08 | 8.37E-11 | 4.51E-06 |
|  |  | **MAX** | 1.20E-01 | 7.45E-02 | 5.05E-02 | 1.72E-02 | 1.66E-01 |
|  |  | **MED** | 1.10E-03 | 6.20E-05 | 1.52E-05 | 7.79E-06 | 4.42E-04 |
| Actives | 50/50 | **AVG** | 0.331 | 0.336 | 0.291 | 0.319 | 0.321 |
|  |  | **MIN** | 0.292 | 0.223 | 0.148 | 0.282 | 0.171 |
|  |  | **MAX** | 0.373 | 0.395 | 0.352 | 0.371 | 0.402 |
|  |  | **MED** | 0.316 | 0.265 | 0.326 | 0.334 | 0.235 |
|  | 100/0 | **AVG** | 0.642 | 0.672 | 0.582 | 0.639 | 0.639 |
|  |  | **MIN** | 0.525 | 0.445 | 0.296 | 0.564 | 0.332 |
|  |  | **MAX** | 0.728 | 0.791 | 0.704 | 0.743 | 0.805 |
|  |  | **MED** | 0.602 | 0.530 | 0.652 | 0.668 | 0.464 |
|  | 0/100 | **AVG** | 3.49E-02 | 6.81E-04 | 1.11E-04 | 4.57E-06 | 3.20E-03 |
|  |  | **MIN** | 4.66E-04 | 1.76E-05 | 8.66E-07 | 9.96E-08 | 2.40E-05 |
|  |  | **MAX** | 6.49E-02 | 4.50E-02 | 6.10E-04 | 4.99E-05 | 1.96E-02 |
|  |  | **MED** | 4.02E-02 | 1.99E-04 | 1.27E-05 | 4.80E-07 | 1.15E-02 |

The ShaEP [8] scores of the top-performing O-LAP models with the training set are shown as average (AVG), minimum (MIN), maximum (MAX), and median (MED) values. The scoring is provided for both inactive decoys and active ligands using the shape only (100/0), ESP only (0/100), and equal weight shape/ESP (50/50) values. Note that equal weight scoring was used in the actual benchmarking.

**Table S9**. Active ligand ranking changes due to docking rescoring using the top docking scoring-based O-LAP models with the test sets.

| **NEU** | | **AA2AR** | | **HSP90** | | **AR** | | **AChE** | |
| --- | --- | --- | --- | --- | --- | --- | --- | --- | --- |
| **CHEMBL** | **Ranking** | **CHEMBL** | **Ranking** | **CHEMBL** | **Ranking** | **CHEMBL** | **Ranking** | **CHEMBL** | **Ranking** |
| 57800 | 92 → 1 | 248299 | 35 → 1 | 393278 | 61 → 1 | 200469 | 322 → 5 | 76173 | 23 → 1 |
| 426810 | 9 → 2 | 275636 | 28 → 2 | 312500 | 227 → 2 | 377958 | 494 → 21 | 1084775 | 26 → 2 |
| 195871 | 4 → 3 | 85864 | 41 → 3 | 302870 | 202 → 3 | 366215 | 514 → 24 | 1173761 | 4 → 3 |
| 136111 | 5 → 4 | 363647 | 4 ↔ 4 | 181571 | 174 → 4 | 386399 | 361 → 31 | 478667 | 13 → 4 |
| 1161284 | 272 → 5 | 1088236 | 74 → 5 | 184313 | 110 → 5 | 399530 | 181 → 35 | 75305 | 37 → 5 |
| 400552 | 2 ← 6 | 1095488 | 44 → 6 | 467888 | 2579 → 7 | 215657 | 277 → 38 | 478666 | 14 → 6 |
| 436250 | 354 → 7 | 1088247 | 152 → 7 | 369509 | 577 → 8 | 426446 | 617 → 41 | 50031 | 6 ← 7 |
| 309192 | 921 → 8 | 274274 | 91 → 8 | 197846 | 163 → 9 | 514748 | 915 → 50 | 1086007 | 8 ↔ 8 |
| 442774 | 24 → 9 | 179644 | 5 ← 9 | 74599 | 483 → 13 | 448474 | 250 → 63 | 225021 | 16 → 9 |
| 22808 | 189 → 10 | 1093480 | 30 → 10 | 367364 | 219 → 14 | 377631 | 135 → 102 | 1172618 | 1 → 10 |
| 311059 | 1086 → 11 | 1088235 | 53 → 12 | 257751 | 1840 → 18 | 467399 | 1391 → 140 | 367067 | 2 → 11 |
| 23009 | 188→ 12 | 471974 | 16 → 13 | 117465 | 520 → 20 | 515048 | 1160 → 211 | 74607 | 36 → 12 |
| 25549 | 26 → 13 | 1087714 | 66 → 14 | 75050 | 143 → 32 | 208239 | 259 → 224 | 449991 | 33 → 13 |
| 350298 | 56 → 14 | 368619 | 88 → 16 | 181722 | 384 → 36 | 207307 | 254 → 226 | 185302 | 51 → 14 |
| 70936 | 37 → 16 | 246262 | 764 → 18 | 359911 | 247 → 45 | 178130 | 485 → 232 | 522286 | 11 → 15 |
| 278852 | 93 → 17 | 481737 | 27 → 21 | 226609 | 825 → 57 | 371915 | 165 → 242 | 175555 | 3 ← 16 |
| 98262 | 711 → 19 | 469876 | 62 → 22 | 207706 | 496 → 68 | 517583 | 1132 → 273 | 225567 | 22 → 17 |
| 301435 | 183 → 20 | 184309 | 143 → 23 | 726 | 130 → 75 | 377371 | 129 → 300 | 597192 | 44 → 18 |
| 81286 | 62 → 21 | 1087820 | 2968 → 24 | 76271 | 3566 → 76 | 457042 | 1375 → 307 | 257126 | 30 → 19 |
| 423292 | 68 → 22 | 183531 | 1 ← 25 | 429842 | 1312 → 88 | 454733 | 1296 → 312 | 370807 | 15 ← 20 |

The O-LAP rescoring could generate higher (→), lower (←) or the same (**↔;** e.g., 363647 for AA2AR or 75050 for AChE**)** ranking for the individual active ligands (CHEMBL codes) as the flexible docking performed with PLANTS. For brevity, only the first 20 top-ranked active ligands are shown.

**Table S10.** Tanimoto similarity analysis between input ligands and the test set compounds at the top 1% of O-LAP docking rescoring results.

| **Ligands ^(1)^** | **Metrics ^(2)^** | **NEU** | **AA2AR** | **HSP90** | **AR** | **AChE** |
| --- | --- | --- | --- | --- | --- | --- |
| Active | **AVG** | 0.21±0.17 | 0.15±0.08 | 0.18±0.20 | 0.07±0.11 | 0.32±0.16 |
|  | **MED** | 0.14 | 0.13 | 0.13 | 0.03 | 0.34 |
|  | **MIN** | 0.04 | 0.01 | 0.00 | 0.00 | 0.04 |
|  | **MAX** | 0.74 | 0.44 | 0.7.0 | 0.50 | 0.72 |
| All | **AVG** | 0.18±0.14 | 0.05±0.03 | 0.03±0.03 | 0.03±0.04 | 0.31±0.16 |
|  | **MED** | 0.12 | 0.04 | 0.02 | 0.02 | 0.33 |
|  | **MIN** | 0.03 | 0.00 | 0.00 | 0.00 | 0.04 |
|  | **MAX** | 0.60 | 0.16 | 0.11 | 0.21 | 0.70 |

The similarity values ≤ 0.4 should indicate that the compared ligand pairs are structurally dissimilar.

^(1)^ Each input ligand (N = 50) was separately compared against the active or all (active and decoy) test set compounds present at the top 1% of the rescored ligands with the top-performing O-LAP model.

^(2)^ The similarity is shown using average (AVG), minimum (MIN), maximum (MAX), and median (MED) values.

**Table S11.** Docking rescoring and rigid docking based on shape-only using the docking scoring-based O-LAP models with the training sets.

| **Shape only O-LAP models in docking rescoring** | | | | | |
| --- | --- | --- | --- | --- | --- |
| **Metrics** | **NEU** | **AA2AR** | **HSP90** | **AR** | **AChE** |
| **AUC** | ***0.97±0.01*** | ***0.79±0.01*** | ***0.78±0.03*** | ***0.81±0.02*** | ***0.86±0.01*** |
| **EFd 0.1%** | ***50*** | ***12.5*** | ***6*** | ***8.6*** | ***27.3*** |
| **EFd 0.5%** | ***74.2*** | ***21.3*** | ***20.9*** | ***18.5*** | ***32*** |
| **EFd 1.0%** | ***83.3*** | **26.4** | ***26.9*** | ***25.8*** | ***39*** |
| **EFd 5.0%** | ***86.4*** | ***38.1*** | ***44.8*** | ***43.7*** | ***64.7*** |
| **BR20** | ***0.85*** | ***0.39*** | ***0.44*** | ***0.43*** | ***0.66*** |
| **N atoms** | 58 | 54 | 45 | 48 | 72 |
| **Rigid docking for Shape only O-LAP models in docking rescoring** | | | | | |
| **AUC** | ***0.97±0.01*** | 0.74±0.02 | ***0.67±0.04*** | ***0.83±0.02*** | ***0.87±0.01*** |
| **EFd 0.1%** | ***15.2*** | *8.2* | ***0*** | ***1.1*** | ***22.3*** |
| **EFd 0.5%** | ***62.1*** | *15.1* | ***3*** | ***14.9*** | ***27.7*** |
| **EFd 1.0%** | ***77.3*** | 19.9 | ***11.9*** | ***23.4*** | ***32.7*** |
| **EFd 5.0%** | ***93.9*** | 33.2 | ***37.3*** | ***37.8*** | ***56.7*** |
| **BR20** | ***0.86*** | 0.34 | ***0.32*** | ***0.38*** | ***0.60*** |
| **N atoms** | 70 | 46 | 61 | 88 | 77 |

The best results, improving or as good as the molecular docking, are shown in bold and italics. Here the EFd 0.1% and 0.5% were calculated for the first time for the original docking results (except for AChE) that were also published previously [7].


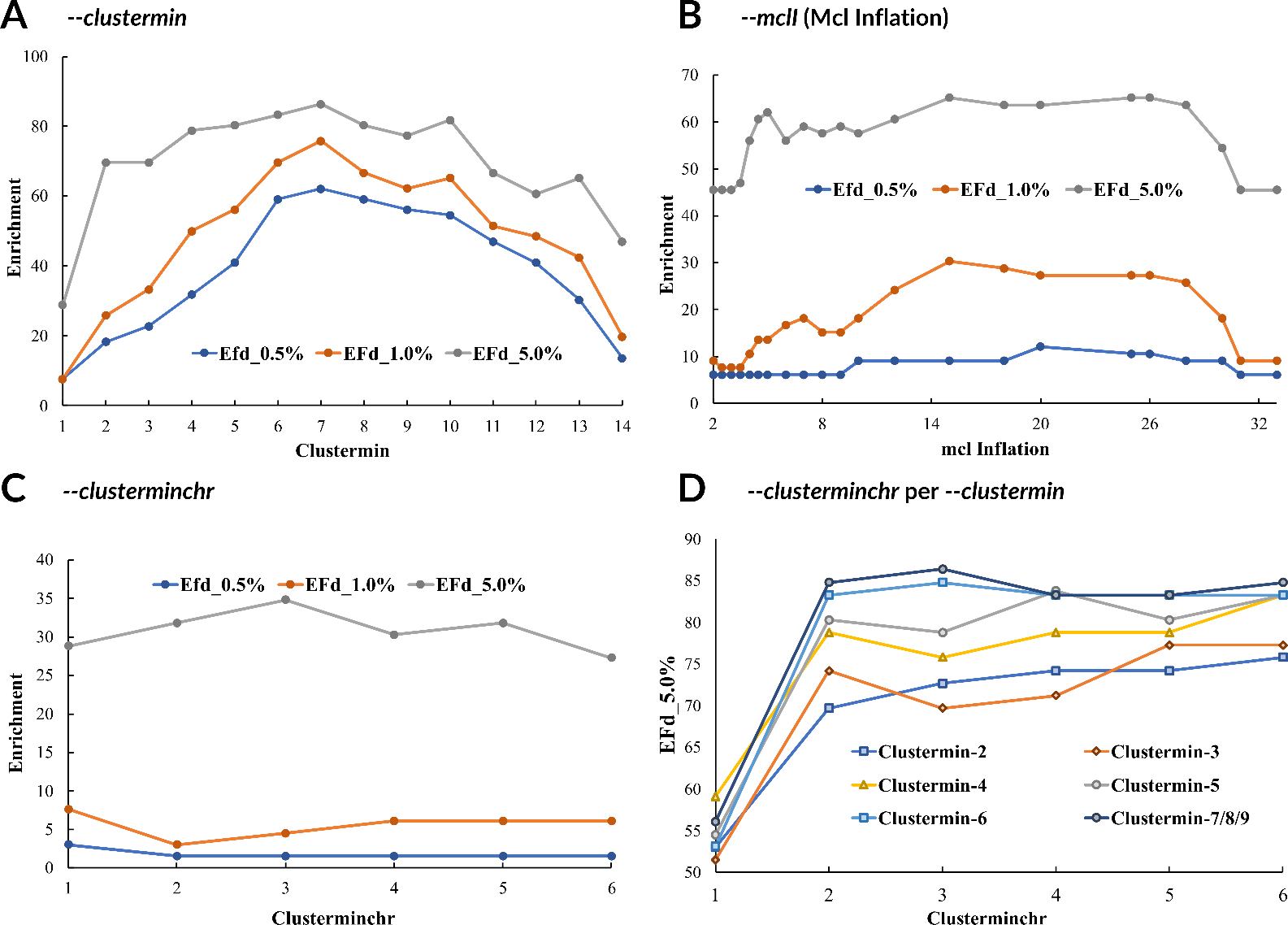


**Fig. S1** The effect of altered O-LAP settings for docking rescoring enrichment with the neuraminidase training set**.** The key O-LAP settings *(--clustermin*, *--clusterminchr*, *--mclI*) are shown altered systematically to generate alternative models for the docking rescoring usage with ShaEP.


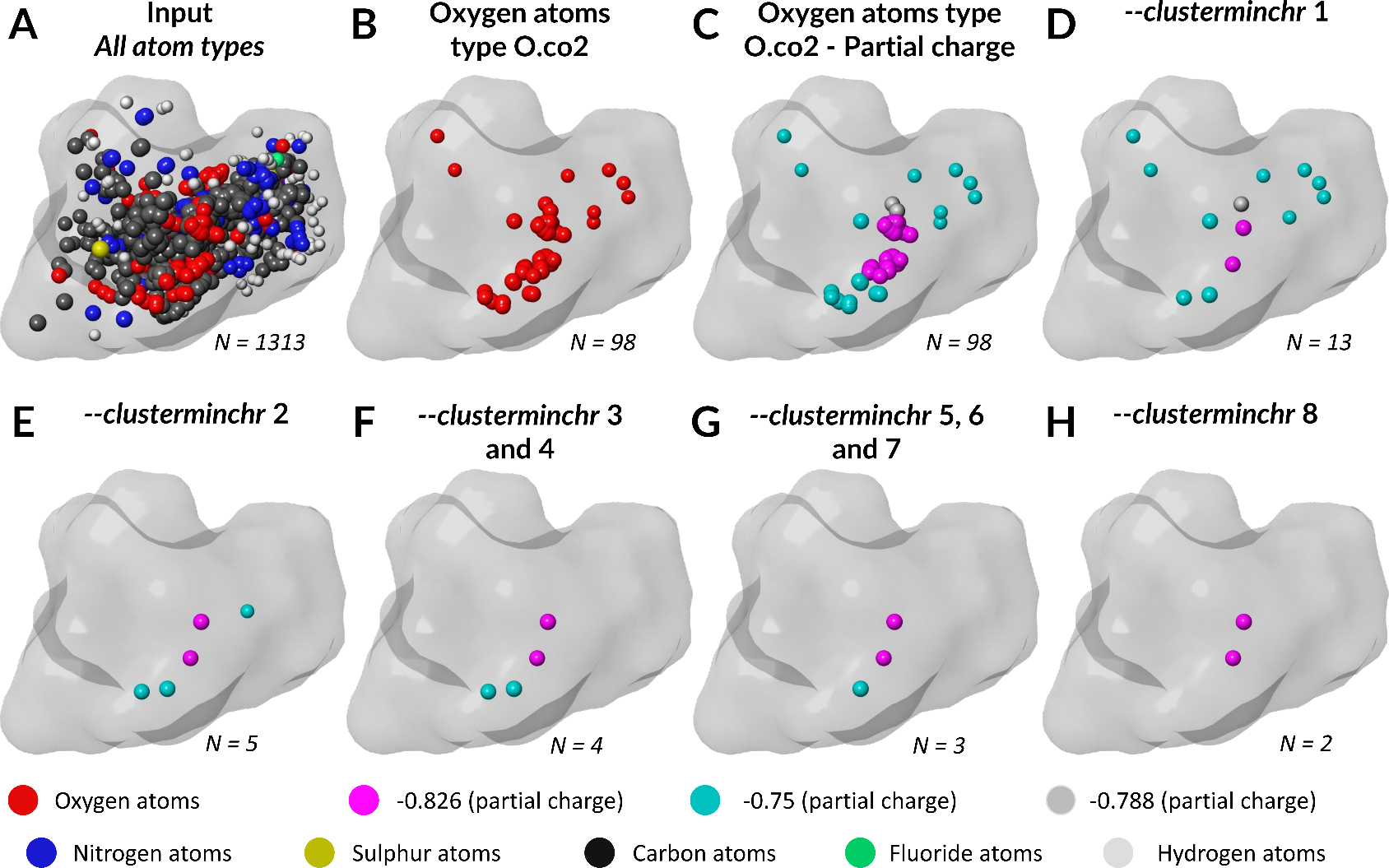


**Fig. S2** The use of the *--clusterminchr* option in the O-LAP modeling with neuraminidase. **A** The input model contains multiple differently colored (N = blue; O = red, S = yellow; C = black, H = white; F = green) and overlapping atoms from 50 top-ranked docked active ligands. **B** The focus is given to the oxygen atoms that belong to the O.co2 Sybyl MOL2 atom type or represent the carboxylate groups. **C** The same Co2 atoms are divided into three categories based on their partial charge (-0.826 = pink; -0.75 = blue; -0.788 = white). Next, it is shown how the clustering changes gradually reduce the amount of O.co2 atoms in the final O-LAP, when different *--clusterminchr* values are applied: **D** 13 atoms with a value of 1; **E** five atoms with a value of 2; **F** four atoms with values of 3 or 4; **G** three atoms with values 5, 6 and 7; and **H** two atoms with a value of 8. The white transparent surface of the input atomic cluster is shown for reference. The default *--nibthreshold* affecting the *--clusteminchr* was used in the example. ￼

**References**

1. Finley JB, Atigadda VR, Duarte F, et al (1999) Novel Aromatic Inhibitors of Influenza Virus Neuraminidase Make Selective Interactions with Conserved Residues and Water Molecules in the Active Site

2. Jaakola VP, Griffith MT, Hanson MA, et al (2008) The 2.6 angstrom crystal structure of a human A2A adenosine receptor bound to an antagonist. Science (1979) 322:1211–1217. https://doi.org/10.1126/science.1164772

3. Zhao D, Xu YM, Cao LQ, et al (2021) Complex Crystal Structure Determination and in vitro Anti–non–small Cell Lung Cancer Activity of Hsp90N Inhibitor SNX-2112. Front Cell Dev Biol 9:. https://doi.org/10.3389/fcell.2021.650106

4. Pereira de Jésus-Tran K, Côté P-L, Cantin L, et al (2006) Comparison of crystal structures of human androgen receptor ligand-binding domain complexed with various agonists reveals molecular determinants responsible for binding affinity. Protein Science 15:987–999. https://doi.org/10.1110/ps.051905906

5. Rydberg EH, Brumshtein B, Greenblatt HM, et al (2006) Complexes of alkylene-linked tacrine dimers with Torpedo californica acetylcholinesterase: Binding of Bis5-tacrine produces a dramatic rearrangement in the active-site gorge. J Med Chem 49:5491–5500. https://doi.org/10.1021/jm060164b

6. Stein RM, Yang Y, Balius TE, et al (2021) Property-Unmatched Decoys in Docking Benchmarks. J Chem Inf Model 61:699–714. https://doi.org/10.1021/acs.jcim.0c00598

7. Vainio MJ, Puranen JS, Johnson MS (2009) ShaEP: Molecular overlay based on shape and electrostatic potential. J Chem Inf Model 49:492–502. https://doi.org/10.1021/ci800315d
